# Elevated expression of *SaMTP8.1* is involved in internal Mn detoxification in the hyperaccumulating ecotype of *Sedum alfredii*

**DOI:** 10.1111/tpj.70240

**Published:** 2025-05-31

**Authors:** Jun Ge, Lingli Lu, Jian Feng Ma

**Affiliations:** ^1^ Institute of Plant Science and Resources Okayama University Chuo 2‐20‐1 Kurashiki 710‐0046 Japan; ^2^ Key Laboratory of Environment Remediation and Ecological Health, Ministry of Education, College of Environmental and Resource Sciences Zhejiang University Hangzhou 310058 China

**Keywords:** *Sedum alfredii*, manganese, tolerance, accumulation, tonoplast, SaMTP8.1, copy number, vacuolar sequestration

## Abstract

*Sedum alfredii* (Sa) is known as a Cd/Zn hyperaccumulator, which usually grows in soil with high Mn in its natural habitat. However, it is unclear how *S. alfredii* copes with high Mn at both physiological and molecular levels. In this study, we characterized the Mn accumulation and tolerance in the hyperaccumulating ecotype (HE) of *S. alfredii* by comparing it with a non‐hyperaccumulating ecotype (NHE). HE and NHE accumulated similar Mn in the leaves after exposure to high Mn, but the young leaves of NHE showed toxicity symptoms (brown spot), whereas no such symptom was observed in HE. Functional characterization of *SaMTP8.1* showed that SaMTP8.1 from both HE and NHE was localized to the tonoplast and showed similar transport activity for Mn in yeast. However, *SaMTP8.1* from HE showed a higher expression level and increased genomic copy number compared with NHE. Ectopic expression of *SaMTP8.1* in rice *osmtp8.1* mutant complemented the mutant phenotype of Mn sensitivity, while overexpression of *SaMTP8.1* in Arabidopsis enhanced tolerance to high Mn. Taken together, our results suggest that higher expression of *SaMTP8.1* is involved in enhanced Mn tolerance through increased vacuolar sequestration of Mn in the leaves of HE *S. alfredii*.

## Significance Statement

A Zn‐/Cd‐hyperaccumulating ecotype of *Sedum alfredii* also shows higher tolerance to Mn in its natural habitat with high Mn concentration; however, the responsible molecular mechanism remains unclear. Here we found that the elevated expression of *SaMTP8.1*, a gene encoding a tonoplast‐localized transporter for Mn, is involved in internal detoxification of high Mn in the leaves by sequestrating Mn into the vacuoles through comparison with a non‐hyperaccumulating ecotype.

## INTRODUCTION

Hyperaccumulators of heavy metals hold great potential for phytoremediation due to their exceptional ability to accumulate these contaminants (Yang et al., 2004). To date, approximately 450 flowering plant species have been identified as hyperaccumulators, including *Sedum alfredii*, a Zn/Cd hyperaccumulator from the Crassulaceae family (Yang et al., 2002, 2004). Another reported Zn/Cd hyperaccumulator, *Sedum plumbizincicola* (Wu et al., 2012), has been suggested to be the same species as *S. alfredii*, though taxonomic discrepancies remain. In its native habitat (mining region), an ecotype of *S. alfredii* is able to accumulate Zn and Cd in the shoots up to 0.5% and 0.1% of the dry weight, respectively, without showing toxicity symptoms (Ge et al., 2021; Yang et al., 2002). Several genes associated with high accumulation and tolerance of Zn and Cd have been functionally characterized in *S. alfredii* in terms of expression pattern, transport activity, subcellular localization, ectopic expression, and so on. For example, the tonoplast‐localized SaHMA3 from a hyperaccumulating ecotype (HE) of *S. alfredii* and SpHMA3 from *S. plumbizincicola* are reported to be involved in vacuolar sequestration of Cd mainly in the shoot for Cd detoxification (Liu et al., 2017; Zhang, Zhang, Shohag, et al., 2016), while SaNramp1 localized to the plasma membrane has been implicated in the partition of Cd and Zn in the shoots of *S. alfredii* (Zhang et al., 2020). *SaZIP4* encodes a plasma membrane‐localized transporter for Zn, which plays an important role in Zn uptake (Yang et al., 2018). On the other hand, three MTP transporters including SaMTP1, SaMTP2h, and SaMTP3h have been implicated in Zn accumulation and tolerance (Zhang et al., 2011, 2021). Through comparing HE with the non‐hyperaccumulating ecotype (NHE) of *S. alfredii*, the expression levels of these transporter genes are generally much higher in HE than in NHE, which have been associated with high accumulation and tolerance of Zn and/or Cd in HE.

In native habitat where HE was found, the soil contains extremely high Mn with total Mn of 11 100 mg kg^−1^ in addition to high Zn and Cd (Ge et al., 2021). The H_2_O‐extracted Mn reached 72.3 mg kg^−1^, which is much higher than that of Cd (0.4 mg kg^−1^) and Zn (37.9 mg kg^−1^). Mineral analysis showed that HE also accumulates high Mn in the shoots (Ge et al., 2021). Exposure to 1 mm Mn resulted in more than 8000 mg kg^−1^ Mn accumulation in the leaves of HE and showed higher Mn tolerance than NHE (Ge et al., 2021). Although Mn is an essential element for plant growth, plants only require about 20–40 mg kg^−1^ Mn of dry weight for healthy growth (Shao et al., 2017). Therefore, HE must have developed some mechanisms for detoxifying high Mn internally. However, the mechanisms underlying Mn tolerance and accumulation are poorly understood in HE.

One of the proposed mechanisms for high Mn tolerance is sequestration of Mn into the vacuoles (Huang et al., 2024; Pittman, 2005; Shao et al., 2017). In some plant species such as rice and Arabidopsis, a member of the cation diffusion facilitator (CDF), MTP8 (metal tolerance protein) has been found to play an important role in internal Mn detoxification by sequestering Mn into the vacuoles. Most members of MTP8 are localized to the tonoplast and show transport activity for Mn. For example, *OsMTP8.1* in rice is required for high Mn tolerance because knockout of this gene resulted in increased Mn sensitivity in shoots, which is characterized by leaf chlorosis (Chen et al., 2013). Similarly, AtMTP8 in Arabidopsis and LaMTP8.1 in *Lupinus albus* are also involved in internal detoxification of high Mn (Eroglu et al., 2016; Olt et al., 2022). However, it is unknown whether the homolog of the MTP8 subgroup in HE, SaMTP8.1, is also required for its high Mn tolerance.

In this study, we physiologically characterized Mn accumulation and tolerance by comparing HE with NHE in hydroponic culture. We then cloned *SaMTP8.1* from both HE and NHE and compared their similarity, expression pattern, transport activity, and subcellular localization. We further ectopically expressed *SaMTP8.1* in rice *osmtp8.1* mutant and Arabidopsis to investigate its role in plants. Our results showed that SaMTP8.1 functions in vacuolar sequestration of Mn in leaves and that its elevated expression level due to multiple genomic copy numbers is involved in the high tolerance of Mn in HE *S. alfredii*.

## RESULTS

### Time‐ and dose‐dependent Mn accumulation in HE and NHE *S. alfredii*

**Figure 1:**
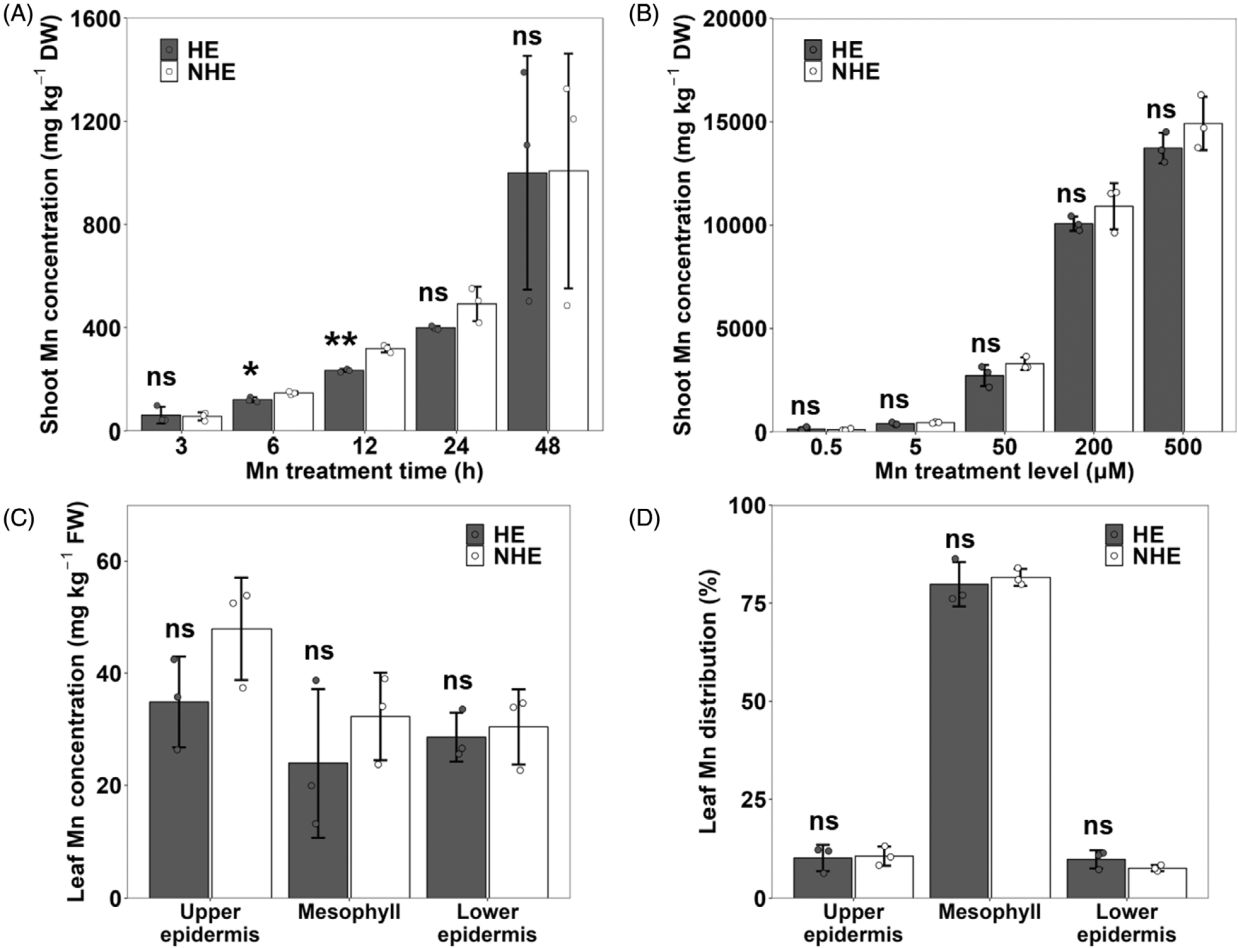
Physiological characterization of Mn accumulation in the shoots of hyperaccumulating ecotype (HE) and non‐hyperaccumulating ecotype (NHE) of *S. alfredii*. (A) Time‐dependent accumulation of Mn in the shoots. Seedlings (20‐day‐old) of HE and NHE *S. alfredii* were exposed to 50 μm Mn for 3, 6, 12, 24, and 48 h. (B) Dose‐dependent accumulation of Mn in the shoots. Seedlings (16‐day‐old) of HE and NHE *S. alfredii* were exposed to different Mn concentrations including 0.5, 5, 50, 200, or 500 μm for 12 days. (C, D) Tissue‐specific accumulation (C) and distribution (D) of Mn in the leaves. Seedlings (18‐day‐old) of HE and NHE *S. alfredii* were exposed to 50 μm Mn for 2 days. Leaves of HE and NHE were dissected into upper epidermis, mesophyll, and lower epidermis. The concentration of Mn (A–C) was determined by ICP‐MS after digestion. The distribution of Mn (D) in the leaves was calculated based on content in each part. Data represent the mean ± SD of three biological replicates (each replicate corresponds to a single plant with three individual plants processed in parallel). Significant differences between HE and NHE are marked: *P < 0.05; **P < 0.01; ns, *P* > 0.05, by Student's *t*‐test. DW, dry weight; FW, fresh weight; ns, not significant.

In natural soil solutions, Mn^2+^ is the most bioavailable form for plant uptake, with concentrations varying from 0.1 to 800 μm depending on soil conditions (Shao et al., 2017). We first compared time‐dependent accumulation of Mn in the shoots of HE and NHE *S. alfredii* under Mn concentrations ranging from 0.5 to 500 μm. HE and NHE *S. alfredii* have been used as hyper‐ and non‐hyper accumulating ecotypes for comparison in many previous studies (e.g. Ge et al., 2021; Zhang et al., 2020). During Mn treatment, the shoot biomass remained unchanged in both HE and NHE due to slow growth and short‐term treatment (Figure S1A). The Mn concentration in the shoots increased with exposure times in both HE and NHE (Figure 1A). After exposure to Mn for a short time (up to 12 h), the shoot Mn concentration was slightly higher in NHE than in HE (Figure 1A). However, at 24 h and later, both ecotypes accumulated similar Mn in the shoots (Figure 1A). At 48 h, the Mn concentration in the shoots reached as high as 965–990 mg kg^−1^ of dry weight in both HE and NHE (Figure 1A). No significant differences in other mineral concentrations, including Mg, P, K, Ca, Fe, Cu, and Zn, were found in either HE or NHE after Mn treatment (Figure S2).

We further compared Mn accumulation in the shoots of two ecotypes exposed to different Mn concentrations. No significant changes in shoot biomass were observed for either HE or NHE during Mn treatment (Figure S1B). The Mn concentration in the shoots increased with increasing Mn supply in the two ecotypes and was similar between the two ecotypes (Figure 1B). At Mn treatment levels higher than 200 μm, both HE and NHE accumulated more than 10 000 mg kg^−1^ DW of Mn in their shoots (Figure 1B). For other mineral elements, there were no consistent differences between different Mn treatment concentrations and different ecotypes (Figure S3), but HE accumulated more Zn than NHE, which is consistent with previous studies (Tian et al., 2009; Yang et al., 2006).

We further analyzed tissue‐specific accumulation of Mn in the leaves. Mn concentration was similar in the upper epidermis, mesophyll, and lower epidermis of both ecotypes (Figure 1C). About 80% Mn was distributed to the mesophyll (Figure 1D), but there was no difference in Mn distribution between HE and NHE. By contrast, Zn concentration was much higher in epidermal cells and HE showed a much higher Zn concentration than NHE (Figure S4G). This result is consistent with a previous report (Tian et al., 2009). Other mineral element concentrations, including Mg, P, K, Ca, Fe, and Cu, showed no large differences between different tissues and ecotypes (Figure S4). Similar to Mn, these mineral elements were primarily distributed to the mesophyll, with no substantial differences between the two ecotypes (Figure S5).

### Comparison of Mn tolerance in HE and NHE *S. alfredii*

**Figure 2:**
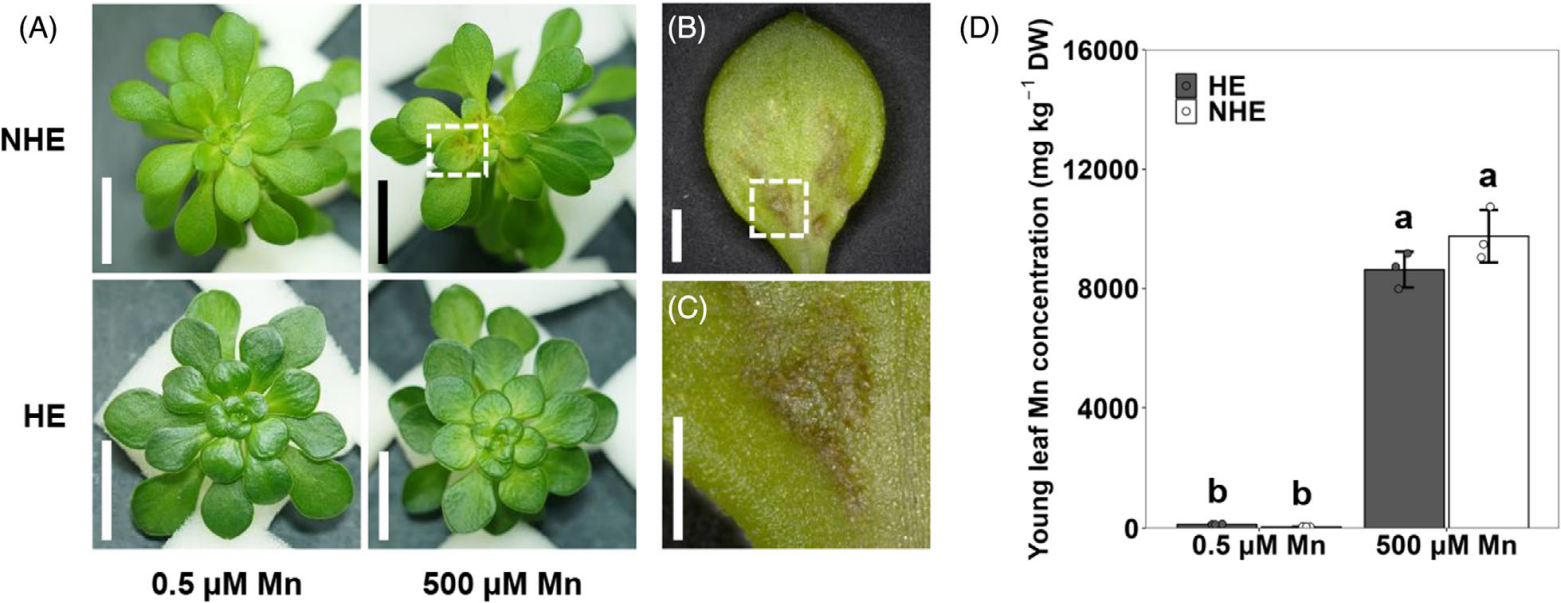
Comparison of Mn tolerance in the shoots of hyperaccumulating ecotype (HE) and non‐hyperaccumulating ecotype (NHE) of *S. alfredii*. (A) Leaf phenotype of HE and NHE *S. alfredii* after 0.5 and 500 μm Mn treatments. Scale bar, 2 cm. (B) Enlarged area of white dashed box in (A). (C) Further enlarged area of white dashed box in (B). Scale bar, 1 mm. (D) Mn concentration in the young leaves of HE and NHE. Seedlings (21‐day‐old) were exposed to 0.5 or 500 μm Mn for 4 days. The concentration of Mn in the young leaves was determined by ICP‐MS after digestion. Data represent the mean ± SD of three biological replicates (each replicate corresponds to a single plant with three individual plants processed in parallel). Significant differences are marked by different letters at *P* < 0.05 using one‐way analysis of variance (anova) followed by Tukey's test. DW, dry weight.

To compare the Mn tolerance between HE and NHE *S. alfredii*, the plants were exposed to 0.5 or 500 μm Mn, and the phenotype was observed daily. There was no difference in the phenotype between HE and NHE exposed to 0.5 μm Mn (Figure 2A). However, after exposure to 500 μm Mn for 4 days, we observed brown spots on the young leaves of NHE *S. alfredii* (Figure 2A–C), but not on the young leaves of HE (Figure 2A), although the shoot biomass was unaffected in both ecotypes (Figure S6). This is due to the slow growth of these ecotypes. After a prolonged exposure time (12 days), the young leaves of HE also showed Mn toxicity symptoms (Figure S7A,C), whereas NHE showed much more severe symptoms by this time (Figure S7A,B). No phenotypic differences were observed between HE and NHE under 0.5 μm Mn for 12 days (Figure S7A).

Analysis of Mn showed that the Mn concentration in the young leaves was similar between HE and NHE after being exposed to 500 μm Mn for 4 days, being 8629–9753 mg kg^−1^ DW (Figure 2D).

### Isolation of *SaMTP8.1* from two ecotypes

To explore the molecular mechanism underlying the high Mn tolerance in HE leaves, we compared the expression level of *MTP* genes including *SaMTP8.1*, *SaMTP8.2*, *SaMTP9*, *SaMTP10*, and *SaMTP11*, since these genes have been reported to be involved in Mn detoxification in other species (Chen et al., 2013; Eroglu et al., 2016; Peiter et al., 2007; Takemoto et al., 2017; Tsunemitsu et al., 2018). A semi‐quantitative PCR analysis showed that among *MTP* members, *SaMTP8.1* exhibited the highest expression level in the shoots of both HE and NHE (Figure S8). Furthermore, the expression level of *SaMTP8.1* in the shoots was much higher in HE than in NHE (Figure S8). This led us to further characterize this gene as described below.

We first cloned *SaMTP8.1* from both ecotypes and compared their similarity. Sequence analysis showed that *SaMTP8.1* from HE and NHE consists of 7 exons and 6 introns. The *SaMTP8.1* from HE and NHE encodes a protein with 422 and 421 amino acid residues, respectively, and they shared 98.10% identity (Figure S9). There are eight residue differences in SaMTP8.1 between the two ecotypes (Figure S9A). SaMTP8.1 from HE and NHE showed 71% similarity to OsMTP8.1 and 63% similarity to AtMTP8, as well as 91% and 93% similarity to their respective SaMTP8.2 (Figure S9). Similar to OsMTP8.1 and AtMTP8, SaMTP8.1 from both ecotypes has 2 DxxxD consensus sequences (x for any amino acid), which were conserved in the Mn‐CDF group close to transmembrane domains 2 and 5 (Montanini et al., 2007), and both of them are predicted to have five transmembrane domains (Figure S9A).

### Expression pattern of *SaMTP8.1* in HE and NHE

**Figure 3:**
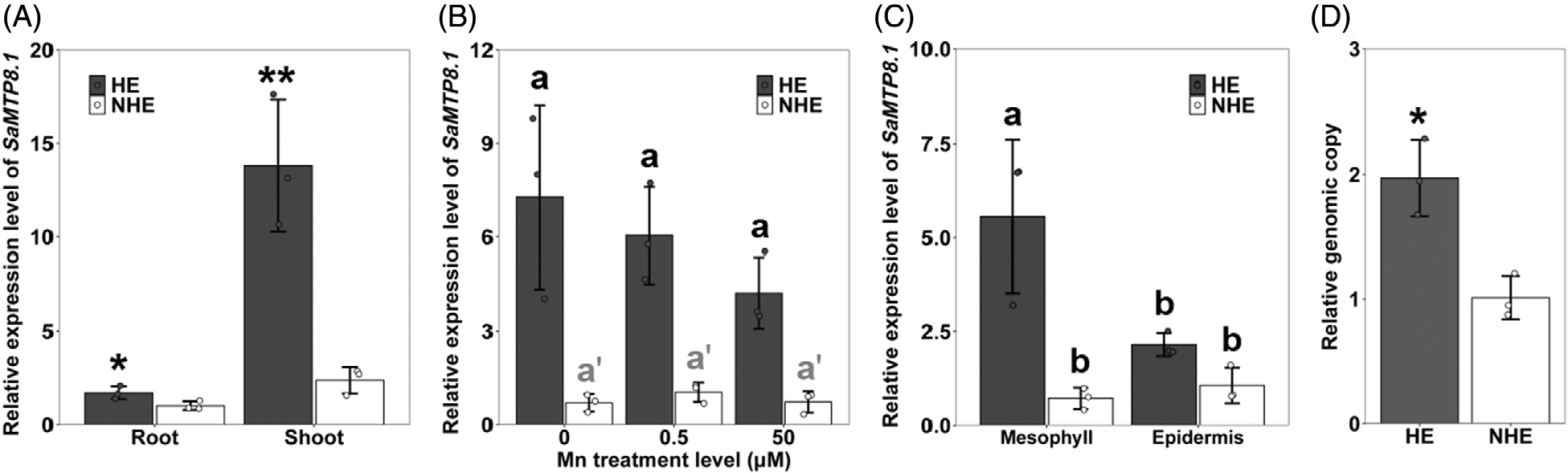
Expression pattern and genomic copy number of *SaMTP8.1* in hyperaccumulating ecotype (HE) and non‐hyperaccumulating ecotype (NHE) of *S. alfredii*. (A) Organ‐dependent expression of *SaMTP8.1* in HE and NHE *S. alfredii*. Roots and shoots of HE and NHE grown in 0.5 μm Mn were sampled for RNA extraction. (B) Response of *SaMTP8.1* in the shoots to different Mn concentrations. Seedlings (19‐day‐old) were exposed to a solution without Mn for 3 days or with 0.5 or 50 μm Mn for 1 day and subjected to RNA extraction. (C) Tissue‐specific expression of *SaMTP8.1* in the leaves of HE and NHE. Seedlings (18‐day‐old) were exposed to 50 μm Mn for 2 days and subjected to RNA extraction. *SaActin1* was used as an internal control. Expression relative to NHE roots at 0.5 μm Mn (A), NHE shoots at 0.5 μm Mn (B), and NHE leaf epidermis (C) is shown. (D) Relative genomic copy number of *SaMTP8.1* in 50 ng gDNA of HE and NHE. The genomic copy number was calculated by relative quantification RT‐PCR, with *SaActin1* as an internal control. Data represent the mean ± SD of three biological replicates: for (A–C), each replicate corresponds to a single plant with three individual plants processed in parallel; and for (D), gDNA was independently extracted from a single plant per replicate with three plants processed in parallel. Significant differences between HE and NHE are marked: *P < 0.05; **P < 0.01, by Student's *t*‐test (A, D), and significant differences are marked by different letters at *P* < 0.05 using one‐way analysis of variance (anova) followed by Tukey's test (B, C).

The expression pattern of *SaMTP8.1* was compared between HE and NHE *S. alfredii*. In both ecotypes, *SaMTP8.1* was more highly expressed in the shoots than in the roots (Figure 3A). However, the expression level in the shoots was much higher in HE than that in NHE (Figure 3A). We also investigated the response of *SaMTP8.1* expression to different Mn supplies in the shoots. Neither Mn deficiency nor excess Mn affected the expression of *SaMTP8.1* in both ecotypes (Figure 3B), indicating that *SaMTP8.1* is constitutively expressed in the shoots of either ecotype. To explore the tissue‐specific expression of *SaMTP8.1* in the leaves of HE and NHE, we also compared its expression between mesophyll and epidermis. In HE, the expression of *SaMTP8.1* was significantly higher in the mesophyll than in the epidermis, whereas that in NHE was similar (Figure 3C). Moreover, the expression level of *SaMTP8.1* in the mesophyll was much higher in HE than in NHE (Figure 3C).

### Genomic copy number of *SaMTP8.1*

To investigate the mechanism underlying higher *SaMTP8.1* expression in HE, we compared its genomic copy number using relative quantitative RT‐PCR. *SaActin1*, a single‐copy gene in both HE and NHE genomes, was used as an internal control. The results showed that the relative genomic copy number of *SaMTP8.1* was doubled in HE compared to NHE (Figure 3D).

### Promoter activity assay of *SaMTP8.1*

We further compared the promoter activities of *SaMTP8.1* between HE and NHE. The putative promoter regions of *SaMTP8.1* from HE and NHE (3276 bp in HE and 2823 bp in NHE) shared 75.36% similarity (Figure S10). Multiple large deletions and single‐nucleotide polymorphisms (SNPs) were observed in the promoter sequences of NHE compared to those of HE, although several small deletions were also observed in HE (Figure S10). To investigate whether these differences affect the expression of *SaMTP8.1*, we fused the promoters from HE and NHE to *GFP* and transiently expressed the constructs in protoplasts isolated from both ecotypes. The promoter activities of *SaMTP8.1* from both ecotypes were detected when expressed in the protoplasts either from HE or NHE (Figure S11). However, there was no significant difference in the relative expression levels of *GFP* (normalized to *DsRed*) between the promoters from the two ecotypes (Figure S12).

### Subcellular localization of SaMTP8.1 from two ecotypes

**Figure 4:**
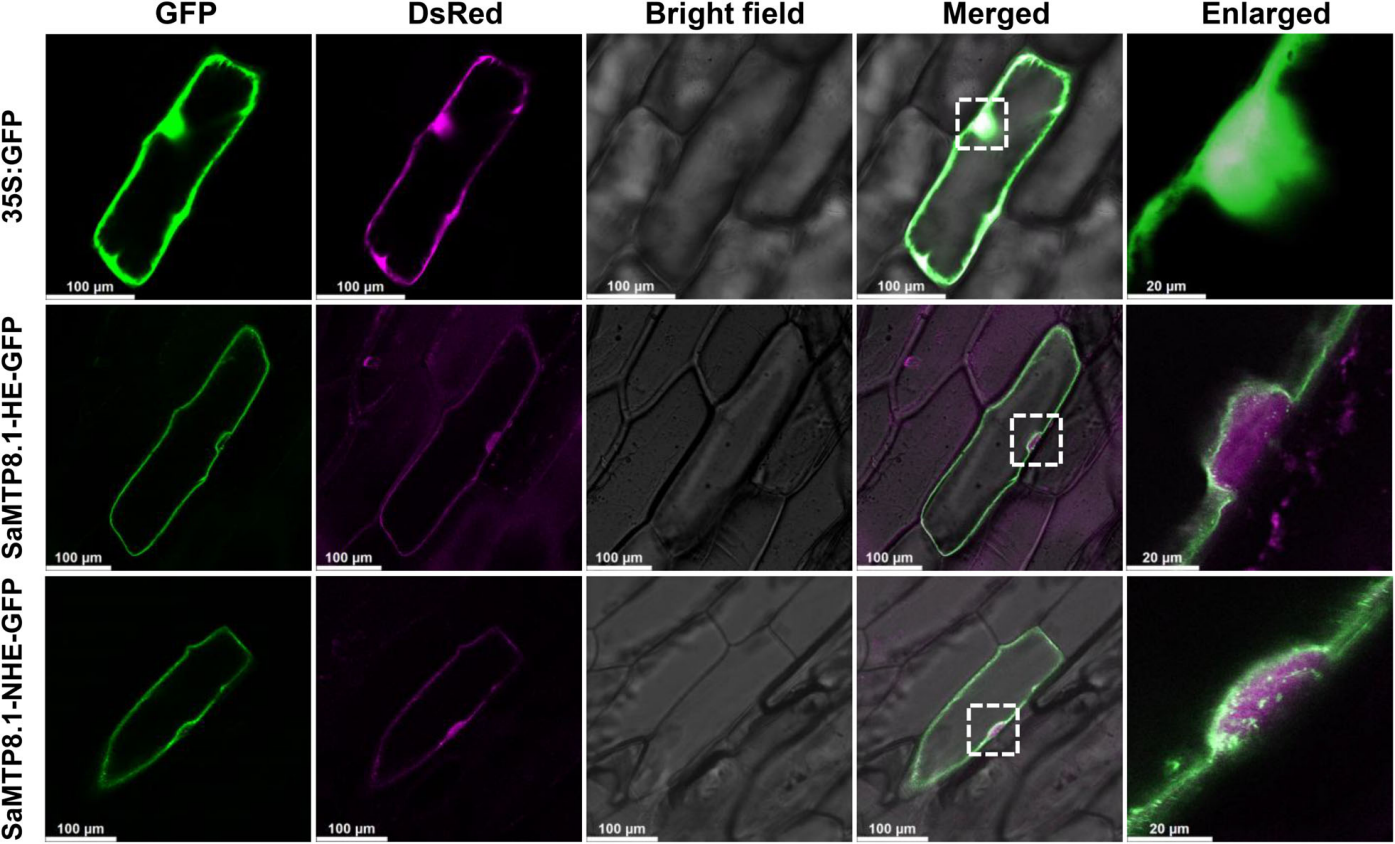
Subcellular localization of SaMTP8.1 from two ecotypes. Plasmids of *SaMTP8.1‐GFP* from two ecotypes or *GFP* alone were transiently expressed in onion epidermal cells with DsRed, a marker of the cytoplasm and nucleus. From left to right: GFP signals, DsRed signals, bright field images, merged images, and enlarged images (magnification of the area of a white dashed box to the left). Scale bar, 100 or 20 μm, as indicated in each sub‐figure.

The subcellular localization of SaMTP8.1 was observed by transiently expressing *SaMTP8.1* fused with GFP from two ecotypes with the cytosol and nucleus marker gene DsRed under the control of the 35S promoter in onion epidermal cells. The GFP signal was colocalized with the DsRed signal when GFP alone was expressed (Figure 4). However, when the *SaMTP8.1‐GFP* fusion from both HE and NHE was expressed, the GFP signal did not overlap with the DsRed signal but was observed at the cell periphery and surrounding the nucleus (Figure 4), indicating that SaMTP8.1 from both HE and NHE was localized to the tonoplast. These results indicate that there was no difference in the subcellular localization of SaMTP8.1 between HE and NHE.

### Transport activity assay of SaMTP8.1 in yeast

**Figure 5:**
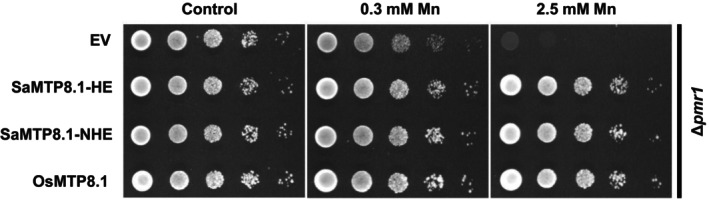
Mn transport activity of SaMTP8.1 from two ecotypes in yeast (*Saccharomyces cerevisiae*). Growth of Mn‐sensitive yeast strain Δ*pmr1* carrying an empty vector (EV), *SaMTP8.1‐HE*, *SaMTP8.1‐NHE*, or *OsMTP8.1* in a SD/−uracil/+galactose solid medium with (0.3 or 2.5 mm Mn) or without additional Mn supply. The plates were incubated at 30°C for 2 days and photographed.

To detect the transport activity of SaMTP8.1 for Mn, a yeast assay was performed using a Mn‐sensitive strain Δ*pmr1*. Without an additional supply of Mn, yeast cells carrying an empty vector (EV), *SaMTP8.1*, or *OsMTP8.1* as a positive control showed no difference in growth (Figure 5). However, in the presence of high Mn (0.3 or 2.5 mm), the growth of yeast cells harboring the empty vector was inhibited, whereas that of cells carrying *SaMTP8.1* or *OsMTP8.1* was hardly affected (Figure 5). Furthermore, SaMTP8.1 from both HE and NHE showed similar growth (Figure 5), indicating that there was no difference in the Mn transport activity of SaMTP8.1 from the two ecotypes.

Because HE *S. alfredii* is known as a Cd/Zn hyperaccumulator, we also investigated the transport activities of SaMTP8.1 for Cd and Zn in yeast mutants. The results showed that the yeast growth did not differ between the empty vector and those expressing *SaMTP8.1* either from HE or NHE at different Cd or Zn concentrations (Figure S13), suggesting that SaMTP8.1 does not have transport activity for Cd and Zn.

### Ectopic expression of *SaMTP8.1* in rice *osmtp8.1* mutant

**Figure 6:**
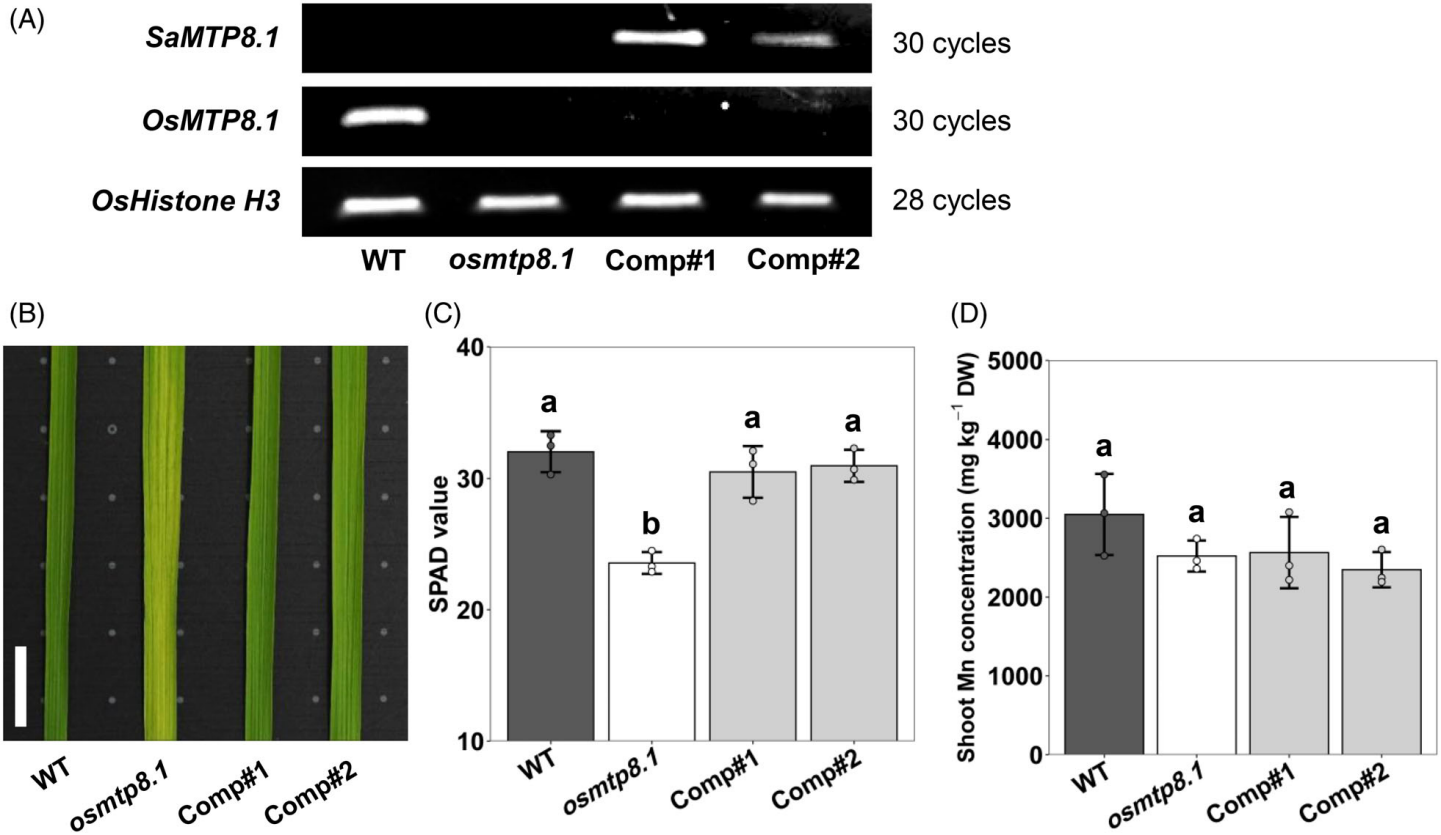
Complementation analysis of *SaMTP8.1* in rice *osmtp8.1* mutant. (A) Expression levels of *SaMTP8.1*, *OsMTP8.1*, and *OsHistone H3* in wild‐type rice (WT, cv. Nipponbare), *osmtp8.1* mutant, and two complementation lines (Comp#1 and Comp#2) of *SaMTP8.1* from HE under the control of the rice *OsMTP8.1* promoter by semi‐quantitative PCR. (B, C) Phenotype of Mn toxicity symptoms (B) and SPAD value (C) of the leaf blades of the newest fully expanded leaf. Scale bar, 1 cm. (D) Concentration of Mn in the shoots. Seedlings (17‐day old) were treated with 500 μm Mn for 4 days. The newest fully expanded leaf blade was used for SPAD value measurement and RNA extraction. *OsHistone H3* was used as an internal control. The concentration of Mn in the shoots was determined by ICP‐MS after digestion. Data represent the mean ± SD of three biological replicates (each replicate corresponds to a single plant with three individual plants processed in parallel). Significant differences are marked by different letters at *P* < 0.05 using one‐way analysis of variance (anova) followed by Tukey's test. DW, dry weight.

Since the stable transformation system of *S*. *alfredii* has not been well established, we were not able to generate *SaMTP8.1* knockout lines to investigate its role in *S. alfredii*. Alternatively, we expressed *SaMTP8.1* from HE in rice *osmtp8.1* mutant (Chen et al., 2013), under the control of *OsMTP8.1* promoter. We selected two independent transgenic lines with high expression of *SaMTP8.1* for phenotypic analysis (Figure 6A). When the plants were supplied with high Mn (500 μm) for 4 days, chlorosis symptoms in the leaf blade of the newest fully expanded leaf were observed in *osmtp8.1* mutant, but not in the WT and two transgenic lines (Figure 6B). The SPAD value of this leaf blade was significantly lower in *osmtp8.1* mutant, but was restored to a similar level as WT in the transgenic lines (Figure 6C). We also compared the Mn (Figure 6D) and other mineral (including Mg, P, K, Ca, Fe, Cu, and Zn, Figure S14) concentrations in the shoots and found that there were no significant differences between these lines. These results indicate that the expression of *SaMTP8.1* in *osmtp8.1* mutant complemented the Mn tolerance phenotype.

### Over‐expression of *SaMTP8.1* in wild‐type Arabidopsis

**Figure 7:**
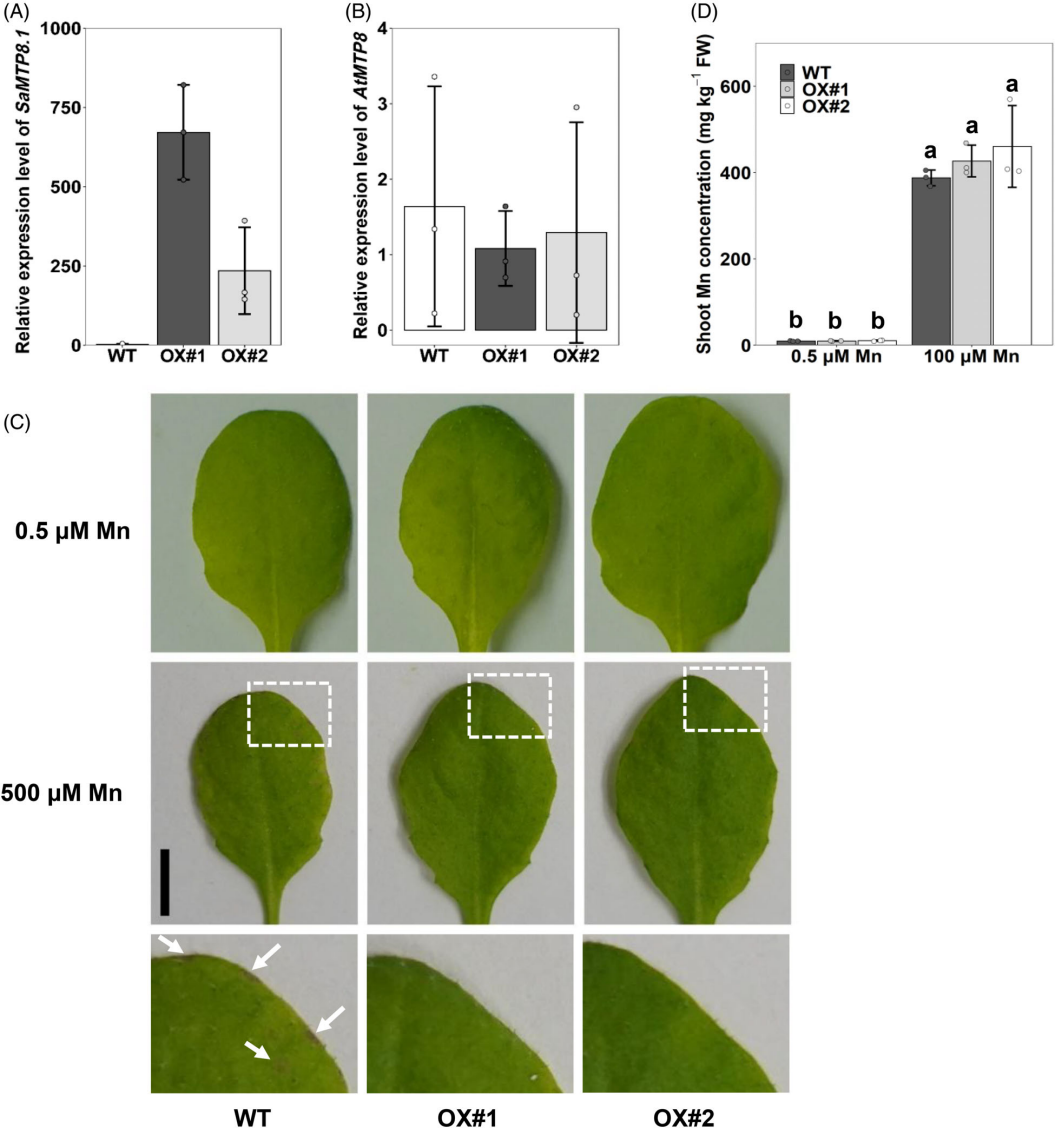
Over‐expression of *SaMTP8.1* enhances Mn tolerance in wild‐type Arabidopsis. (A, B) Expression levels of *SaMTP8.1* (A) and *AtMTP8* (B) in wild‐type Arabidopsis Col‐0 (WT) and two over‐expression lines (OX#1 and OX#2) of *SaMTP8.1* from HE under the control of its own promoter. (C) Phenotype of Mn toxicity symptoms in leaves. Scale bar, 5 mm. The white dashed boxes indicate the magnified area at the bottom. The white arrows indicate the symptoms of Mn toxicity. (D) Concentration of Mn in the shoots. The plants were treated with 0.5 or 100 μm Mn for 10 days. Leaves with 100 μm Mn treatment were collected for RNA extraction. *AtActin* was used as an internal control. Expression relative to WT is shown. The concentration of Mn in the shoots was determined by ICP‐MS after digestion. Data represent the mean ± SD of three biological replicates (each replicate corresponds to a single plant with three individual plants processed in parallel). Significant differences are marked by different letters at *P* < 0.05 using one‐way analysis of variance (anova) followed by Tukey's test. FW, fresh weight.

We further over‐expressed *SaMTP8.1* from HE in the wild‐type Arabidopsis under the control of its own promoter, to confirm its role in Mn tolerance in planta. We selected two independent lines with high expression of *SaMTP8.1* (Figure 7A). These lines showed a similar expression level of endogenous *AtMTP8* as the WT (Figure 7B). When grown at normal Mn concentration (0.5 μm), the leaf phenotype was similar between the WT and over‐expression lines (Figure 7C). However, when grown in the presence of 100 μm Mn for 10 days, brown spots, the typical Mn toxicity symptom, were observed in the leaves of WT but not in the over‐expression lines (Figure 7C). The shoot Mn (Figure 7D) and other mineral (including Mg, P, K, Ca, Fe, Cu, and Zn, Figure S15) concentrations were similar between these lines. These results show that the expression of *SaMTP8.1* enhanced Mn tolerance in Arabidopsis.

## DISCUSSION

Since the discovery of the hyperaccumulating ecotype (HE) of *S. alfredii* as a Zn/Cd hyperaccumulator, extensive studies have been made on the physiological and molecular mechanisms of its high accumulation and tolerance of Zn/Cd through comparisons with its non‐hyperaccumulating ecotype (NHE) (Lu et al., 2013; Tian et al., 2009, 2011, 2017; Yang et al., 2002, 2004). However, although in the natural habitat of *S. alfredii*, Mn concentration in soil was higher than Zn and Cd (Ge et al., 2021), the mechanisms underlying tolerance and accumulation of Mn are poorly understood. In the present study, through comparing two ecotypes collected from different locations with high and low Mn in soil, we found that HE is able to tolerate high Mn by sequestrating it into the vacuoles mainly in the leaf mesophylls (Figure 1D). We further found that SaMTP8.1, a tonoplast‐localized Mn transporter, mediated vacuolar sequestration of Mn (Figure 4 and 5), and that the elevated expression of *SaMTP8.1* (Figure 3) contributes to high Mn tolerance in HE.

### Characteristics of Mn accumulation and tolerance in *S. alfredii*

Physiological analysis showed that the Mn accumulation in the shoots was similar between HE and NHE after exposure to high Mn (Figure 1A–C). There was also no difference in Mn distribution between epidermal cells and mesophylls (Figure 1D). This accumulation characteristic is different from that of Zn and Cd because HE usually accumulates much higher Zn and Cd than NHE (Lu et al., 2013; Tian et al., 2009, 2011). However, HE and NHE differed in their tolerance to high Mn. In contrast to HE, which did not show toxicity symptoms in the young leaves after exposure to high Mn for 4 days, typical Mn toxicity symptoms were observed in NHE (Figure 2A–C). Since HE and NHE showed similar Mn levels in the young leaves (Figure 2D), these findings indicate that HE has developed a mechanism for detoxifying internal high Mn, which is probably associated with vacuolar sequestration of Mn mediated by SaMTP8.1 as discussed below.

### Elevated expression of *SaMTP8.1* contributes to high Mn tolerance in HE *S. alfredii*

Sequestration of heavy metals into the vacuoles is a common strategy for internal detoxification (Verbruggen et al., 2009). For instance, Cd and Zn in rice are sequestered into root vacuoles through the tonoplast‐localized transporter OsHMA3 (Ueno et al., 2010). Vacuolar sequestration of Cd and Zn by SaHMA3 and SpHMA3 is also reported to be vital for the hypertolerance of HE *S. alfredii* and *S. plumbizincicola* (Liu et al., 2017; Zhang, Zhang, Shohag, et al., 2016). Furthermore, a tonoplast‐localized SaCAX2 is implicated in vacuolar sequestration of Mn in *S. alfredii*, although its exact role is not understood (Zhang, Zhang, Lu, et al., 2016). In the present study, we found that SaMTP8.1 is involved in internal detoxification of Mn in *S. alfredii*. This is supported by several lines of evidence. Firstly, *SaMTP8.1* is the most abundantly expressed gene among the *MTP* members in the HE shoot (Figure S8). Furthermore, the higher expression was found in the mesophylls (Figure 3C), which is consistent with high Mn accumulation in these tissues (Figure 1D). Secondly, SaMTP8.1 is localized to the tonoplast (Figure 4) and enhances Mn tolerance when expressed in yeast (Figure 5). Thirdly, ectopic expression of *SaMTP8.1* in rice *osmtp8.1* mutant complemented its phenotype of Mn sensitivity (Figure 6). Fourthly, over‐expression of *SaMTP8.1* in Arabidopsis enhanced its tolerance to high Mn (Figure 7). Homologous genes of *MTP8.1* have also been associated with Mn detoxification in other plant species including rice, Arabidopsis, *Lupinus albus*, *Pyrus bretschneideri* Rehd, and *Glycine max* (Chen et al., 2013; Eroglu et al., 2016; Li, Dong, et al., 2021; Li, Zheng, et al., 2021; Olt et al., 2022), indicating that MTP8.1‐mediated Mn detoxification is conserved across plant species.

Functional analysis of SaMTP8.1 showed that there were no differences in subcellular localization and transport activity in yeast between SaMTP8.1 from two ecotypes differing in Mn tolerance (Figures 4 and 5). These results indicate that SaMTP8.1 from both HE and NHE has a similar function in sequestering Mn into the vacuoles. However, the expression levels of *SaMTP8.1* were much higher in HE shoots and leaf mesophyll than those in NHE (Figure 3A–C). This elevated expression of *SaMTP8.1* probably contributes to high Mn tolerance in HE. A similar phenomenon was observed in other metal hyperaccumulators. For example, in a Cd‐hyperaccumulating ecotype of *Noccaea caerulescens* (formerly *Thlaspi caerulescens*), elevated expression of *TcHMA3* is crucial for its high Cd tolerance through vacuolar sequestration of Cd (Ueno et al., 2011). Higher expression of *SaHMA3* in HE *S. alfredii* and *SpHMA3* in *S. plumbizincicola* was also reported to be involved in high Cd tolerance (Liu et al., 2017; Zhang, Zhang, Shohag, et al., 2016), while higher expression of *SpHMA1* was reported to be required for detoxification of Cd in the chloroplast in *S. plumbizincicola* (Zhao et al., 2019). Recently, *SpAMT1;2* in *S. plumbizincicola* has also shown higher expression in the hyperaccumulating ecotype and plays a role in the interaction between NH_4_^+^ and Cd uptake (Yu et al., 2024). In another Cd hyperaccumulator, *Arabidopsis halleri*, an increased expression of *HMA4* is required for high tolerance and accumulation of Zn and Cd (Hanikenne et al., 2008). These findings indicate that elevated expression of genes related to hypertolerance or hyperaccumulation of heavy metals seems a result of long‐term adaptation to extreme metallic environments (Verbruggen et al., 2009).

The enhanced expression level of these tolerance genes has been attributed to multiple genomic copy numbers such as *TcHMA3*, *SaHMA3*, and *SaMTP8.1* and/or cis‐element regulation such as *AhHMA4*, *SpHMA1*, and *SpAMT1;2* (Hanikenne et al., 2008; Ueno et al., 2011; Yu et al., 2024; Zhang, Zhang, Shohag, et al., 2016; Zhao et al., 2019). In the present study, we found that the genomic copy number of *SaMTP8.1* was greater in HE than that in NHE (Figure 3D). However, the copy number is not proportional to the gene expression level (Figure 3A–C) as the expression level increased more than the genomic copy number in HE compared with NHE. This finding suggests that there are other unknown mechanisms regulating the expression of *SaMTP8.1* in HE in addition to the genomic copy number. Although we compared the promoter activity between two ecotypes, it is unlikely that this is related to the elevated expression of *SaMTP8.1* in HE (Figure S12). Therefore, the exact mechanisms underlying higher expression of *SaMTP8.1* in HE remain to be further examined in the future.

In conclusion, our results indicate that the high Mn tolerance in the shoots of HE *S. alfredii* is achieved by vacuolar sequestration of Mn. Furthermore, the elevated expression of *SaMTP8.1*, a tonoplast‐localized Mn transporter gene, is at least partially attributable to increased genomic copy number and contributes to the high Mn tolerance observed in HE *S. alfredii*.

## EXPERIMENTAL PROCEDURES

### Plant materials and growth conditions

A hyperaccumulating ecotype (HE) of *Sedum alfredii* was originally obtained from an old Pb/Zn mining region (Quzhou City, Zhejiang Province, China), while a non‐hyperaccumulating ecotype (NHE) was collected from a tea plantation (Hangzhou City, Zhejiang Province, China) (Yang et al., 2002, 2004). They show contrasting accumulation in Cd and Zn in the shoots (Lu et al., 2013; Tian et al., 2009, 2011). The seeds of the two ecotypes of *S. alfredii* were germinated on a mixture of perlite and vermiculite. The uniform branches about 2–3 cm in length were cut and hydroponically cultured in 1/10 strength Hoagland solution containing the macronutrients (mm); 0.5 KNO_3_, 0.5 Ca(NO_3_)_2_, 0.2 MgSO_4_, and 0.1 (NH_4_)H_2_PO_4_, and micronutrients (μm); 0.5 MnSO_4_, 0.2 CuSO_4_, 0.4 ZnSO_4_, 3 H_3_BO_3_, 1 (NH_4_)_6_Mo_7_O_24_, and 20 FeNa‐EDTA. The pH of the solution was adjusted to 6.0 by 1 N NaOH. The nutrient solution was continuously aerated and renewed every 2 days. The plants were grown in an environmentally controlled growth room with day/night temperatures of 22°C/20°C under natural light.

For most experiments, each biological replicate corresponded to a single plant, with three individual plants processed in parallel. For genomic copy number analysis, gDNA was independently extracted from a single plant per replicate, with three plants processed in parallel. As for the promoter activity assay, protoplasts were isolated from stems of six individual plants, pooled together, and evenly divided into three samples for independent transformations.

### Physiological characterization of Mn accumulation

To investigate time‐dependent accumulation of Mn, seedlings (20‐day‐old) of both HE and NHE *S. alfredii* were pre‐cultured in the 1/10 Hoagland solution containing 0.05 μm Mn, followed by exposure to 50 μm Mn. The shoots were harvested at different time points (3, 6, 12, 24, and 48 h).

In a dose–response experiment, seedlings (16‐day‐old) of both HE and NHE were exposed to a solution containing different Mn concentrations, including 0.5, 5, 50, 200, or 500 μm. After 12 days, the shoots were harvested.

To analyze the tissue‐specific accumulation of Mn in leaves, seedlings (18‐day‐old) of both HE and NHE pre‐cultured in 1/10 Hoagland solution with 0.05 μm Mn were exposed to 50 μm Mn for 2 days. The leaves were dissected into the upper epidermis, mesophyll, and lower epidermis using tweezers and subjected to Mn determination and RNA extraction as described below.

### Comparison of Mn tolerance between HE and NHE in the shoots

To compare the Mn tolerance between HE and NHE, seedlings (21‐day‐old) of both HE and NHE pre‐cultured in 1/10 Hoagland solution with 0.05 μm Mn were exposed to a nutrient solution containing 0.5 or 500 μm Mn. After 4 days, the plants were photographed and the young leaves were harvested. The phenotype of Mn toxicity symptoms in NHE young leaves was observed using a VHX‐6000 stereomicroscope (Keyence Corporation, Osaka, Japan). After 12 days, the plants were photographed again.

### Expression pattern of Mn‐related *MTPs* by semi‐quantitative PCR

To investigate the expression pattern of Mn‐related *MTPs*, including *SaMTP8.1*, *SaMTP8.2*, *SaMTP9*, *SaMTP10*, and *SaMTP11* in HE and NHE shoots, semi‐quantitative PCR was performed. The seedlings (21‐day‐old) of both HE and NHE were cultured normally with 0.5 μm Mn. The shoots were then harvested for RNA extraction using the Quick‐RNA Miniprep Kit (Zymo Research Corp., Irvine, CA, USA), followed by conversion to cDNA using the ReverTra Ace qPCR RT Master Mix with gDNA remover (TOYOBO Co., Ltd., Osaka, Japan). The expression levels of the above genes were determined by semi‐quantitative PCR using the Quick Taq® HS DyeMix (TOYOBO Co., Ltd., Osaka, Japan). *SaActin1* was used as an internal control (Zhang, Zhang, Shohag, et al., 2016). The primers were designed based on transcriptome databases (Ge et al., 2022), and the sequence information is listed in Table S1.

### Cloning of *SaMTP8.1* and similarity analysis

The cDNA containing the full‐length open‐reading frame (ORF) of *SaMTP8.1* from both ecotypes was amplified by KOD FX Neo (TOYOBO Co., Ltd., Osaka, Japan) using primers listed in Table S1, which were designed based on the sequences in transcriptome databases (Ge et al., 2022). The PCR fragments were subcloned into the pTA2 vector (TOYOBO Co., Ltd., Osaka, Japan) and sequenced using a Big‐Dye sequencing kit (Applied Biosystems, Foster City, CA, USA) on an Applied Biosystems 3130 Genetic Analyzer (Applied Biosystems, Foster City, CA, USA). Protein transmembrane domains were predicted using the TMHMM server (https://services.healthtech.dtu.dk/services/TMHMM‐2.0/).

Alignment of SaMTP8.1 from HE and NHE with OsMTP8.1 and AtMTP8 was performed using DNAMAN (Lynnon Biosoft, San Ramon, CA, USA). Phylogenetic analysis was conducted by MEGA X (MEGA Software, Tempe, AZ, USA) using the maximum likelihood method with 1000 bootstraps.

### Expression patterns of *SaMTP8.1* by real‐time RT‐PCR

To examine the expression pattern of *SaMTP8.1*, seedlings (19‐day‐old) of both HE and NHE were pre‐cultured in 1/10 Hoagland solution, followed by exposure to a nutrient solution without Mn for 3 days or with 0.5 or 50 μm Mn for 1 day. The roots and shoots were then separately harvested.

The total RNA was extracted from different organs (roots and shoots) and different tissues (epidermal cells and mesophylls), followed by reverse transcription as described above. Expression levels of *SaMTP8.1* from two ecotypes were determined by quantitative RT‐PCR method using the KOD SYBR™ qPCR Mix (TOYOBO Co., Ltd., Osaka, Japanc) on a real‐time PCR machine CFX96 (Bio‐Rad, Hercules, CA, USA). *SaActin1* was used as an internal control (Zhang, Zhang, Shohag, et al., 2016). Primers used are listed in Table S1. Relative expression level was calculated using ΔΔC_T_ method.

### Determination of genomic copy number of *SaMTP8.1*

The genomic DNA was extracted from the leaves of HE and NHE using a modified CTAB method (Murray & Thompson, 1980) and 50 ng gDNA was used as a template. The relative genomic copy number of *SaMTP8.1* was determined by the quantitative RT‐PCR method using the KOD SYBR™ qPCR Mix (TOYOBO Co., Ltd., Osaka, Japan) on a real‐time PCR machine CFX96 (Bio‐Rad, Hercules, CA, USA). *SaActin1*, which is a single‐copy gene in both HE and NHE genomes, was used as an internal control. The genomic copy number of *SaMTP8.1* was normalized based on the cycle threshold value for *SaActin1*. Primers are listed in Table S1.

### Promoter activity assay of *SaMTP8.1*

The putative promoter regions of *SaMTP8.1* from HE and NHE (a 3276 bp region upstream of the translational start codon in HE and 2823 bp in NHE) were amplified from their gDNA using the primers listed in Table S1. The fragments amplified were cloned into the *EcoR*I and *Sal*I sites of the modified pCAMBIA1300‐GFP vector carrying GFP and NOS terminator by In‐Fusion Snap Assembly Master Mix (Takara Bio Inc., Shiga, Japan). These constructs, along with DsRed (Clontech Laboratories, San Jose, CA, USA) driven by the 35S promoter as an internal control (Lei et al., 2020), were transformed into the stem protoplasts isolated from HE and NHE using a polyethylene glycol (PEG)‐mediated method, modified from Gao et al. (2017) and Zhao et al. (2019).

Briefly, young stems of HE and NHE were cut into 0.5–1 mm segments and immersed in a digestion solution (1.5% [w/v] Cellulase Onozuka RS [Yakult Honsha Co., Ltd., Tokyo, Japan], 0.4% [w/v] Macerozyme Onozuka R‐10 [Yakult Honsha Co., Ltd., Tokyo, Japan], 0.4 m mannitol, 20 mm KCl, 10 mm CaCl_2_, 0.1% BSA, 50 mg L^−1^ carbenicillin, 20 mm MES, pH = 5.7). The samples were incubated in the dark at 30°C with gentle shaking at 40 rpm for 2 h. The digestion solution was filtered through a 40 μm mesh and centrifuged at 100 ***g*** for 5 min to collect protoplasts. The sedimented protoplasts were washed with W5 solution (154 mm NaCl, 125 mm CaCl_2_, 5 mm KCl, 5 mm glucose, 2 mm MES, pH = 5.7) and resuspended in MMg solution (0.4 m mannitol, 15 mm MgCl_2_, 4 mm MES, pH = 5.7). For plasmid transformation, 5 μg constructed *GFP* plasmid, 5 μg *DsRed* plasmid, and 110 μl PEG solution (0.4 m mannitol, 0.1 m CaCl_2_, 40% PEG 3350) were added to 100 μl protoplast solution and incubated in the dark at 25°C for 15 min. Subsequently, 1 ml W5 solution was added, and the protoplasts were centrifuged at 100 ***g*** for 5 min. The pellet was resuspended in WI solution (0.6 m mannitol, 4 mm KCl, 4 mm MES, pH = 5.7) and incubated in the dark at 25°C for 18 h.

The fluorescence signals of GFP and DsRed were observed with a confocal laser scanning microscope (TCS SP8x; Leica Microsystems GmbH, Wetzlar, Germany). The protoplasts were collected for RNA extraction, followed by reverse transcription as described above. The expression levels of *GFP* and *DsRed* were determined by quantitative RT‐PCR method using the KOD SYBR™ qPCR Mix (TOYOBO Co., Ltd., Osaka, Japanc) on a real‐time PCR machine CFX96 (Bio‐Rad, Hercules, CA, USA). The relative expression of *GFP* was normalized to *DsRed*. Primers are listed in Table S1.

### Subcellular localization of SaMTP8.1

The ORF of *SaMTP8.1* from HE and NHE without a translational stop codon was amplified using the primers listed in Table S1. The fragments amplified were cloned into the *Kpn*I and *Sal*I sites of the modified pCAMBIA1300‐GFP vector carrying the 35S promoter and NOS terminator by In‐Fusion Snap Assembly Master Mix (Takara Bio Inc., Shiga, Japan). Gold particles (1 μm) coated with *GFP* as a control or *SaMTP8.1‐GFP* constructs from two ecotypes along with DsRed (Clontech Laboratories, San Jose, CA, USA) as a cytosolic and nuclear marker were delivered into onion epidermis cells using particle bombardment (Helios Gene Gun system; Bio‐Rad, Hercules, CA, USA; Yamaji et al., 2016). The fluorescence signal was observed with a confocal laser scanning microscope (TCS SP8x; Leica Microsystems GmbH, Wetzlar, Germany) after incubation in the dark at room temperature for 16 h.

### Transport activity assay in yeast

To test the Mn/Cd/Zn transport activities of SaMTP8.1 in yeast (*Saccharomyces cerevisiae*), the ORF of *SaMTP8.1* from HE and NHE was amplified using the primers listed in Table S1, followed by cloning into the *EcoR*I and *Xba*I sites of the pYES2 vector (Thermo Fisher Scientific, Waltham, MA, USA) under the control of a galactose‐inducible promoter by In‐Fusion Snap Assembly Master Mix (Takara Bio Inc., Shiga, Japan). OsMTP8.1 was used as a positive control for Mn transport activity as reported before (Chen et al., 2013). The pYES2 empty vector, pYES2‐*SaMTP8.1*, and pYES2‐*OsMTP8.1* were respectively transformed into the Mn‐sensitive mutant Δ*pmr1* (Mat a; his3Δ1; leu2Δ0; met15Δ0; ura3Δ0; PMR1::kanMX4) which lacks the Golgi‐localized Ca/Mn‐ATPase PMR1 responsible for Mn tolerance in yeast (Antebi & Fink, 1992). For the Cd and Zn transport activity assay, a Cd‐sensitive mutant Δ*ycf1* (Mat a; his3Δ1; leu2Δ0; met15Δ0; ura3Δ0; YCF1::kanMX4) which lacks the Ycf1 protein that encodes an MgATP‐energized glutathione *S*‐conjugate transporter for Cd detoxification in vacuoles (Li et al., 1997), and a Zn‐sensitive mutant Δ*zrc1/cot1* (Mat a; his3Δ1; leu2Δ0; met15Δ0; ura3Δ0; ZRC1::natMX; COT1::kanMX4) which lacks Zrc1 and Cot1 proteins for Zn detoxification in vacuoles (MacDiarmid et al., 2003) were used with a modified PEG/LiAc/ssDNA method (Gietz & Schiestl, 2007).

To examine the transport activities for Mn/Cd/Zn, Δ*pmr1*, Δ*ycf1*, and Δ*zrc1/cot1* expressing empty vector, *SaMTP8.1*, or *OsMTP8.1* were pre‐cultured in SD/−uracil/+glucose liquid medium until the OD_600_ reached 1.0. Ten‐fold serial dilutions of each strain were prepared, and 5 μl of each dilution was spotted on SD/−uracil/+galactose solid medium with different metal supplies (0, 0.3, and 2.5 mm Mn; 0, 10, and 20 μm Cd; or 0, 200, and 500 μm Zn). After incubating at 30°C for 2 days, the plates were photographed.

### Ectopic expression of *SaMTP8.1* in rice *osmtp8.1* mutant

Since the stable transgenic system for *S*. *alfredii* has not been well established, we ectopically expressed *SaMTP8.1* from HE into rice *osmtp8.1* mutant to investigate its role in planta. The promoter fragment of *OsMTP8.1* (a 2954 bp region upstream of the translational start codon of *OsMTP8.1*) and cDNA fragment of *SaMTP8.1* were amplified from gDNA of Nipponbare and cDNA of HE *S. alfredii* using the primers listed in Table S1, respectively. These two fragments were cloned into the modified pPZP2H‐lac vector carrying NOS terminator by In‐Fusion Snap Assembly Master Mix (Takara Bio Inc., Shiga, Japan) using *Kpn*I and *Hind*III restriction sites. The construction was transformed into calluses of *osmtp8.1* mutant reported before (Chen et al., 2013) by *Agrobacterium tumefaciens*‐mediated transformation (Hiei et al., 1994). Two independent lines (T_1_ generation) were selected for further analysis as described below.

To detect the expression of *SaMTP8.1* in the transgenic rice lines, the leaf blade was sampled for RNA extraction by RNeasy plant mini kit (Qiagen N.V., Venlo, The Netherlands) followed by cDNA synthesis according to the manufacturer of ReverTra Ace qPCR RT Master Mix with gDNA remover (TOYOBO Co., Ltd., Osaka, Japan). The expression levels of *SaMTP8.1* and *OsMTP8.1* were determined by semi‐quantitative PCR using the Quick Taq® HS DyeMix (TOYOBO Co., Ltd., Osaka, Japan). *OsHistone H3* was used as an internal control. Primers are listed in Table S1.

To test the Mn tolerance, the wild‐type rice (cv. Nipponbare), *osmtp8.1* mutant, and two independent complementation lines were exposed to a solution containing 500 μm Mn. After 4 days, the newest fully expanded leaf blade was subjected to the measurement of SPAD value by SPAD‐5 chlorophyll meter (Konika Minolta, Tokyo, Japan) and photographed. The shoots were harvested and subjected to mineral determination as described below.

### Over‐expression of *SaMTP8.1* in wild‐type Arabidopsis

To further confirm the function of *SaMTP8.1* in planta, we also over‐expressed *SaMTP8.1* from HE in Arabidopsis under the control of its own promoter. To do this, a 5366 bp genomic fragment including the 3276 bp promoter region of *SaMTP8.1* (upstream of the translational start codon) and the 2090 bp coding region of *SaMTP8.1* was amplified from gDNA of HE *S. alfredii* and inserted into the modified pPZP2H‐lac vector carrying NOS terminator by In‐Fusion Snap Assembly Master Mix (Takara Bio Inc., Shiga, Japan) using *Kpn*I and *Hind*III restriction sites. The primers used are listed in Table S1. The construction was transformed into wild‐type Arabidopsis (*Col*‐0) by *Agrobacterium tumefaciens*‐mediated floral dip method. Two independent lines (T_2_ generation) were selected and used for further analysis.

To detect the expression of *SaMTP8.1* in the transgenic Arabidopsis lines, leaf samples were taken for RNA extraction as described above. The expression levels of *SaMTP8.1* and *AtMTP8* were determined by KOD SYBR™ qPCR Mix (TOYOBO Co., Ltd., Osaka, Japan) on a real‐time PCR machine CFX96 (Bio‐Rad, Hercules, CA, USA). *AtActin* was used as an internal control. Primers are listed in Table S1.

To compare the Mn tolerance, the WT and two transgenic lines were exposed to a solution containing 0.5 or 100 μm Mn. After 10 days, the phenotype of the leaves was observed and photographed. Subsequently, the shoots were harvested and subjected to mineral determination as described below.

### Determination of mineral element concentrations

Plant samples harvested as described above were dried at 70°C for at least 2 days, and then digested by 2 ml 61% HNO_3_ (w/v) at a temperature of up to 135°C. The concentrations of Mn and other minerals (including Mg, P, K, Ca, Fe, Cu, and Zn) in the digestion solution were determined with inductively coupled plasma‐mass spectrometry (ICP‐MS 7700X; Agilent Technologies, Santa Clara, CA, USA).

### Statistical analysis

Statistics was performed with Student's *t*‐test or one‐way analysis of variance (anova) followed by a Tukey's test in SPSS Statistics 20 (IBM Corp., Armonk, NY, USA). The significance of differences was defined as: ns for not significant, *P < 0.05, **P < 0.01, and different letters for *P* < 0.05.

## ACCESSION NUMBERS

Sequence data from this article can be found in the GenBank under accession numbers PQ416765 (SaMTP8.1‐HE) and PQ416766 (SaMTP8.1‐NHE).

## AUTHOR CONTRIBUTIONS

JG and JFM conceived, designed, and performed the experiments. LL commented on this work. JG and JFM analyzed the data and wrote the article.

## CONFLICT OF INTEREST

The authors declare no conflict of interest.

## Supporting information


**Table S1.** List of primer sequences.
**Figure S1.** Time‐ and dose‐dependent changes in shoot biomass of hyperaccumulating ecotype (HE) and non‐hyperaccumulating ecotype (NHE) of *S. alfredii* under Mn treatment. (A) Time‐dependent changes in shoot biomass. Seedlings (20‐day‐old) of HE and NHE *S. alfredii* were exposed to 50 μm Mn for 3, 6, 12, 24, and 48 h. Shoot fresh weight at 3 h was defined as 100% for HE and NHE, respectively. (B) Dose‐dependent changes in shoot biomass. Seedlings (16‐day‐old) of HE and NHE *S. alfredii* were exposed to different Mn concentrations including 0.5, 5, 50, 200, or 500 μm for 12 days. Shoot fresh weight in the presence of 0.5 μm Mn treatment was defined as 100% for HE and NHE, respectively. Data represent the mean ± SD of three biological replicates (each replicate corresponds to a single plant with three individual plants processed in parallel). Significant differences are marked by different letters at *P* < 0.05 using one‐way analysis of variance (anova) followed by Tukey's test. FW, fresh weight.
**Figure S2.** Time‐dependent accumulation of mineral elements (Mg, P, K, Ca, Fe, Cu, and Zn) in the shoots of hyperaccumulating ecotype (HE) and non‐hyperaccumulating ecotype (NHE) of *S. alfredii*. Seedlings (20‐day‐old) of HE and NHE *S. alfredii* were exposed to 50 μm Mn for 3, 6, 12, 24, and 48 h. The concentrations of Mg (A), P (B), K (C), Ca (D), Fe (E), Cu (F), and Zn (G) in the shoots were determined by ICP‐MS after digestion. Data represent the mean ± SD of three biological replicates (each replicate corresponds to a single plant with three individual plants processed in parallel). Significant differences are marked by different letters at *P* < 0.05 using one‐way analysis of variance (anova) followed by Tukey's test. DW, dry weight.
**Figure S3.** Dose‐dependent accumulation of mineral elements (Mg, P, K, Ca, Fe, Cu, and Zn) in the shoots of hyperaccumulating ecotype (HE) and non‐hyperaccumulating ecotype (NHE) of *S. alfredii*. Seedlings (16‐day‐old) of HE and NHE *S. alfredii* were exposed to different Mn concentrations including 0.5, 5, 50, 200, or 500 μm for 12 days. The concentrations of Mg (A), P (B), K (C), Ca (D), Fe (E), Cu (F), and Zn (G) in the shoots were determined by ICP‐MS after digestion. Data represent the mean ± SD of three biological replicates (each replicate corresponds to a single plant with three individual plants processed in parallel). Significant differences are marked by different letters at *P* < 0.05 using one‐way analysis of variance (anova) followed by Tukey's test. DW, dry weight.
**Figure S4.** Tissue‐specific accumulation of mineral elements (Mg, P, K, Ca, Fe, Cu, and Zn) in the leaves of hyperaccumulating ecotype (HE) and non‐hyperaccumulating ecotype (NHE) of *S. alfredii*. Seedlings (18‐day‐old) of HE and NHE *S. alfredii* were exposed to 50 μm Mn for 2 days. Leaves of HE and NHE were dissected into upper epidermis, mesophyll, and lower epidermis. The concentrations of Mg (A), P (B), K (C), Ca (D), Fe (E), Cu (F), and Zn (G) in the leaves were determined by ICP‐MS after digestion. Data represent the mean ± SD of three biological replicates (each replicate corresponds to a single plant with three individual plants processed in parallel). Significant differences between HE and NHE are marked: *P < 0.05; **P < 0.01; ns, *P* > 0.05, by Student's *t*‐test. FW, fresh weight.
**Figure S5.** Tissue‐specific distribution of mineral elements (Mg, P, K, Ca, Fe, Cu, and Zn) in the leaves of hyperaccumulating ecotype (HE) and non‐hyperaccumulating ecotype (NHE) of *S. alfredii*. Seedlings (18‐day‐old) of HE and NHE *S. alfredii* were exposed to 50 μm Mn for 2 days. Leaves of HE and NHE were dissected into upper epidermis, mesophyll, and lower epidermis. The distributions of Mg (A), P (B), K (C), Ca (D), Fe (E), Cu (F), and Zn (G) in the leaves were calculated based on content in each part. Data represent the mean ± SD of three biological replicates (each replicate corresponds to a single plant with three individual plants processed in parallel). Significant differences between HE and NHE are marked: *P < 0.05; **P < 0.01; ns, *P* > 0.05, by Student's *t*‐test.
**Figure S6.** Changes in shoot biomass of hyperaccumulating ecotype (HE) and non‐hyperaccumulating ecotype (NHE) of *S. alfredii* under Mn treatment. Seedlings (21‐day‐old) of HE and NHE *S. alfredii* were exposed to 0.5 or 500 μm Mn for 4 days. The shoot biomass of HE and NHE under 0.5 μm Mn treatment was defined as 100%, respectively. Data represent the mean ± SD of three biological replicates (each replicate corresponds to a single plant with three individual plants processed in parallel). Significant differences are marked by different letters at *P* < 0.05 using one‐way analysis of variance (anova) followed by Tukey's test. DW, dry weight.
**Figure S7.** Comparison of Mn tolerance in the shoots of hyperaccumulating ecotype (HE) and non‐hyperaccumulating ecotype (NHE) of *S. alfredii* with prolonged exposure. (A) Leaf phenotype of HE and NHE *S. alfredii* at 0.5 and 500 μm Mn. (B, C) Enlarged area of white dashed box in (a) of NHE (B) and HE (C). Scale bar, 2 cm. Seedlings (21‐day‐old) were exposed to 0.5 or 500 μm Mn for 12 days. The white arrows indicate the symptoms of Mn toxicity.
**Figure S8.** Semi‐quantification of Mn‐related *MTPs* expression in the shoots of hyperaccumulating ecotype (HE) and non‐hyperaccumulating ecotype (NHE) of *S. alfredii*. Seedlings (21‐day‐old) of HE and NHE *S. alfredii* were cultured in a solution with 0.5 μm Mn. The shoots of HE and NHE were collected for RNA extraction. The expression levels of *SaMTP8.1*, *SaMTP8.2*, *SaMTP9*, *SaMTP10*, and *SaMTP11* were analyzed by semi‐quantitative PCR. *SaActin1* was used as an internal control.
**Figure S9.** Alignment and phylogenetic analysis of SaMTP8.1. (A) Protein sequence alignment of SaMTP8.1‐HE, SaMTP8.1‐NHE, SaMTP8.2‐HE, SaMTP8.2‐NHE, OsMTP8.1, and AtMTP8. Shading indicates identical (black) or similar (pink for 75% and blue for 50%) amino acid residues. The transmembrane domains of SaMTP8.1‐HE are predicted by TMHMM and are marked in blue boxes. The DxxxD consensus sequence (x for any amino acid) which were conserved in Mn‐CDF group close to transmembrane domains 2 and 5 (Montanini et al., 2007) was marked in red boxes. TM, transmembrane domain. (B) Phylogenetic analysis of the MTP family protein sequences from HE *S. alfredii*, NHE *S. alfredii*, rice, and Arabidopsis. Red text indicates MTP8.1 members from HE and NHE *S. alfredii*. Blue text indicates MTP8.1 from rice and MTP8 from Arabidopsis. Gray text indicates protein IDs in the respective databases.
**Figure S10.** Alignment of putative *SaMTP8.1* promoter regions from two ecotypes. Sequence comparison of putative promoter regions between HE and NHE. These regions correspond to 3276 and 2823 bp upstream of the translational start codon of *SaMTP8.1* in HE and NHE, respectively. Red letters indicate nucleotide differences between the two ecotypes.
**Figure S11.** Expression of p*SaMTP8.1‐GFP* and p*35S‐DsRed* in HE and NHE protoplasts. Protoplasts isolated from HE and NHE were co‐transformed with p*SaMTP8.1‐HE‐GFP* or p*SaMTP8.1‐NHE‐GFP* along with p3*5S‐DsRed* as an internal control. From left to right: GFP signals, DsRed signals, bright field images, and merged images. Scale bar, 10 μm.
**Figure S12.** Promoter activity of *SaMTP8.1* in hyperaccumulating ecotype (HE) and non‐hyperaccumulating ecotype (NHE) of *S. alfredii*. *GFP* driven by the putative *SaMTP8.1* promoter from HE and NHE was introduced into protoplasts isolated from HE and NHE, with p*35S‐DsRed* as an internal control. The expression of *GFP* was normalized to *DsRed*. Expression relative to p*SaMTP8.1‐NHE‐GFP* in NHE protoplast was shown. Protoplasts were isolated from stems of six individual plants of each ecotype, pooled, and evenly divided into three samples for independent transformations. Data represent the mean ± SD of three biological replicates. Significant differences are marked by different letters at *P* < 0.05 using one‐way analysis of variance (anova) followed by Tukey's test.
**Figure S13.** Cd and Zn transport activities of SaMTP8.1 from two ecotypes in yeast (*Saccharomyces cerevisiae*). (A) Growth of Cd‐sensitive yeast strain Δ*ycf1* carrying an empty vector (EV), *SaMTP8.1‐HE*, *SaMTP8.1‐NHE*, or *OsMTP8.1* in a SD/−uracil/+galactose solid medium with (5 or 10 μm Cd) or without additional Cd supply. (B) Growth of Zn‐sensitive yeast strain Δ*zrc1/cot1* carrying an empty vector (EV), *SaMTP8.1‐HE*, *SaMTP8.1‐NHE*, or *OsMTP8.1* in a SD/−uracil/+galactose solid medium with (200 or 500 μm Zn) or without additional Zn supply. The plates were incubated at 30°C for 2 days and photographed.
**Figure S14.** Effect of *SaMTP8.1* complementation in rice *osmtp8.1* mutant on mineral (Mg, P, K, Ca, Fe, Cu, and Zn) accumulation. Concentrations of Mg (A), P (B), K (C), Ca (D), Fe (E), Cu (F), and Zn (G) in the shoots of wild‐type rice (WT, cv. Nipponbare), *osmtp8.1* mutant, and two complementation lines (Comp#1 and Comp#2) of *SaMTP8.1* from HE under the control of rice *OsMTP8.1* promoter. Seedlings (17‐day old) were treated with 500 μm Mn for 4 days. The mineral concentrations were determined by ICP‐MS after digestion. Data represent the mean ± SD of three biological replicates (each replicate corresponds to a single plant with three individual plants processed in parallel). Significant differences are marked by different letters at *P* < 0.05 using one‐way analysis of variance (anova) followed by Tukey's test. DW, dry weight.
**Figure S15.** Effect of *SaMTP8.1* overexpression in wild‐type Arabidopsis on mineral (Mg, P, K, Ca, Fe, Cu, and Zn) accumulation. Concentrations of Mg (A), P (B), K (C), Ca (D), Fe (E), Cu (F), and Zn (G) in the shoots of wild‐type Arabidopsis Col‐0 (WT) and two overexpression lines (OX#1 and OX#2) of *SaMTP8.1* from HE under the control of its own promoter. The plants were treated with 0.5 or 100 μm Mn for 10 days. The mineral concentrations were determined by ICP‐MS after digestion. Data represent the mean ± SD of three biological replicates (each replicate corresponds to a single plant with three individual plants processed in parallel). Significant differences are marked by different letters at *P* < 0.05 using one‐way analysis of variance (anova) followed by Tukey's test. FW, fresh weight.

## Data Availability

All relevant data can be found within the manuscript and its supporting materials.
