# Supplementary material for: Elevated expression of SaMTP8.1 is involved in internal Mn detoxification in the hyperaccumulating ecotype of Sedum alfredii
Source: Plant J. 2025 May 31;122(5):e70240. doi: 10.1111/tpj.70240 (PMC12126605; doi:10.1111/tpj.70240)
Supplement: Supplementary file 1 — Table S1. List of primer sequences. Figure S1. Time‐ and dose‐dependent changes in shoot biomass of hyperaccumulating ecotype (HE) and non‐hyperaccumulating ecotype (NHE) of S. alfredii under Mn treatment. (A) Time‐dependent changes in shoot biomass. Seedlings (20‐day‐old) of HE and NHE S. alfredii were exposed to 50 μm Mn for 3, 6, 12, 24, and 48 h. Shoot fresh weight at 3 h was defined as 100% for HE and NHE, respectively. (B) Dose‐dependent changes in shoot biomass. Seedlings (16‐day‐old) of HE and NHE S. alfredii were exposed to different Mn concentrations including 0.5, 5, 50, 200, or 500 μm for 12 days. Shoot fresh weight in the presence of 0.5 μm Mn treatment was defined as 100% for HE and NHE, respectively. Data represent the mean ± SD of three biological replicates (each replicate corresponds to a single plant with three individual plants processed in parallel). Significant differences are marked by different letters at P < 0.05 using one‐way analysis of variance (anova) followed by Tukey's test. FW, fresh weight. Figure S2. Time‐dependent accumulation of mineral elements (Mg, P, K, Ca, Fe, Cu, and Zn) in the shoots of hyperaccumulating ecotype (HE) and non‐hyperaccumulating ecotype (NHE) of S. alfredii. Seedlings (20‐day‐old) of HE and NHE S. alfredii were exposed to 50 μm Mn for 3, 6, 12, 24, and 48 h. The concentrations of Mg (A), P (B), K (C), Ca (D), Fe (E), Cu (F), and Zn (G) in the shoots were determined by ICP‐MS after digestion. Data represent the mean ± SD of three biological replicates (each replicate corresponds to a single plant with three individual plants processed in parallel). Significant differences are marked by different letters at P < 0.05 using one‐way analysis of variance (anova) followed by Tukey's test. DW, dry weight. Figure S3. Dose‐dependent accumulation of mineral elements (Mg, P, K, Ca, Fe, Cu, and Zn) in the shoots of hyperaccumulating ecotype (HE) and non‐hyperaccumulating ecotype (NHE) of S. alfredii. Seedlings (16‐day‐old) [file TPJ-122-0-s001.pptx]

## Slide 1
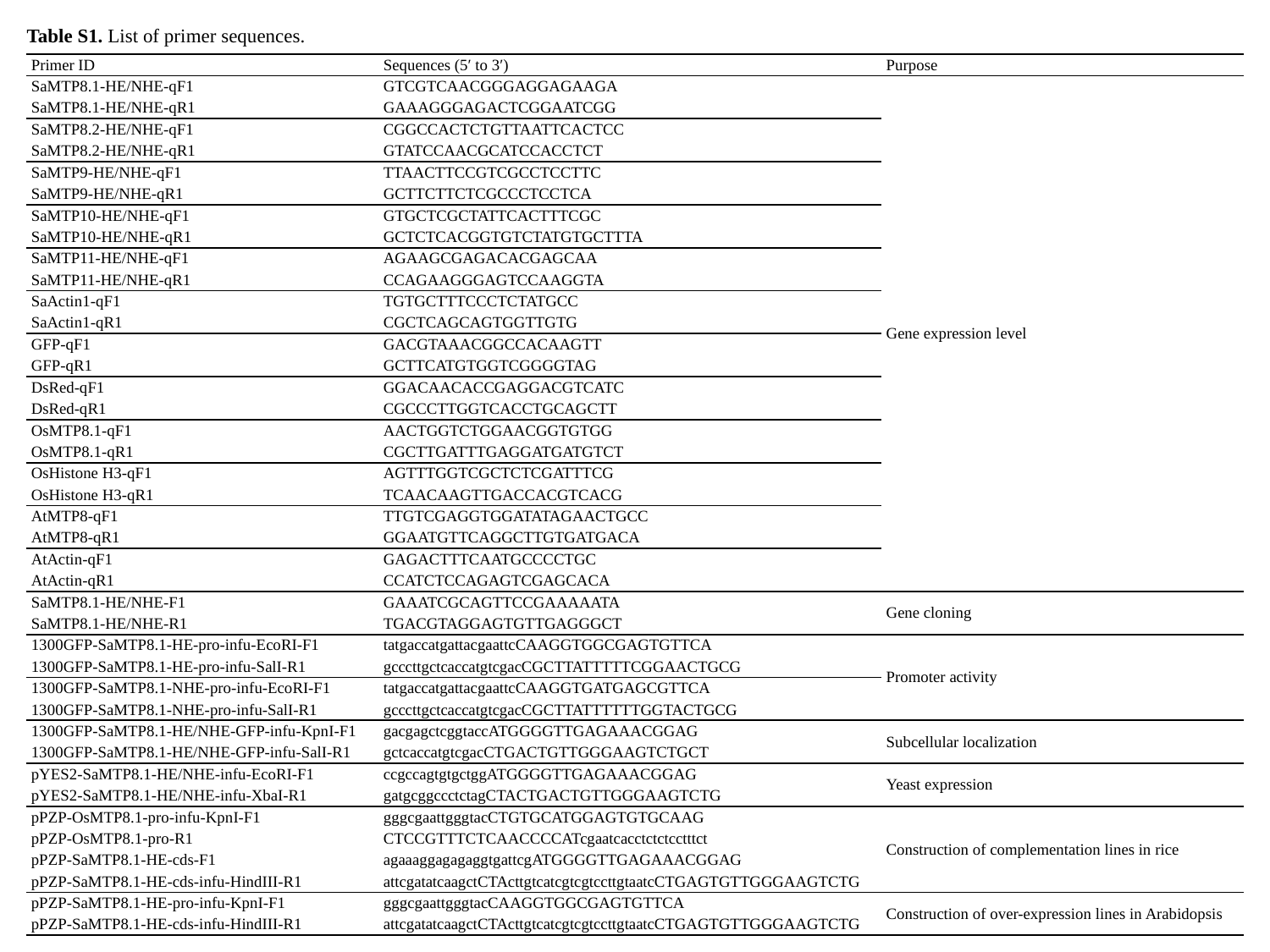

Table S1. List of primer sequences.
| Primer ID | Sequences (5′ to 3′) | Purpose |
| --- | --- | --- |
| SaMTP8.1-HE/NHE-qF1 | GTCGTCAACGGGAGGAGAAGA | Gene expression level |
| SaMTP8.1-HE/NHE-qR1 | GAAAGGGAGACTCGGAATCGG | |
| SaMTP8.2-HE/NHE-qF1 | CGGCCACTCTGTTAATTCACTCC | |
| SaMTP8.2-HE/NHE-qR1 | GTATCCAACGCATCCACCTCT | |
| SaMTP9-HE/NHE-qF1 | TTAACTTCCGTCGCCTCCTTC | |
| SaMTP9-HE/NHE-qR1 | GCTTCTTCTCGCCCTCCTCA | |
| SaMTP10-HE/NHE-qF1 | GTGCTCGCTATTCACTTTCGC | |
| SaMTP10-HE/NHE-qR1 | GCTCTCACGGTGTCTATGTGCTTTA | |
| SaMTP11-HE/NHE-qF1 | AGAAGCGAGACACGAGCAA | |
| SaMTP11-HE/NHE-qR1 | CCAGAAGGGAGTCCAAGGTA | |
| SaActin1-qF1 | TGTGCTTTCCCTCTATGCC | |
| SaActin1-qR1 | CGCTCAGCAGTGGTTGTG | |
| GFP-qF1 | GACGTAAACGGCCACAAGTT | |
| GFP-qR1 | GCTTCATGTGGTCGGGGTAG | |
| DsRed-qF1 | GGACAACACCGAGGACGTCATC | |
| DsRed-qR1 | CGCCCTTGGTCACCTGCAGCTT | |
| OsMTP8.1-qF1 | AACTGGTCTGGAACGGTGTGG | |
| OsMTP8.1-qR1 | CGCTTGATTTGAGGATGATGTCT | |
| OsHistone H3-qF1 | AGTTTGGTCGCTCTCGATTTCG | |
| OsHistone H3-qR1 | TCAACAAGTTGACCACGTCACG | |
| AtMTP8-qF1 | TTGTCGAGGTGGATATAGAACTGCC | |
| AtMTP8-qR1 | GGAATGTTCAGGCTTGTGATGACA | |
| AtActin-qF1 | GAGACTTTCAATGCCCCTGC | |
| AtActin-qR1 | CCATCTCCAGAGTCGAGCACA | |
| SaMTP8.1-HE/NHE-F1 | GAAATCGCAGTTCCGAAAAATA | Gene cloning |
| SaMTP8.1-HE/NHE-R1 | TGACGTAGGAGTGTTGAGGGCT | |
| 1300GFP-SaMTP8.1-HE-pro-infu-EcoRI-F1 | tatgaccatgattacgaattcCAAGGTGGCGAGTGTTCA | Promoter activity |
| 1300GFP-SaMTP8.1-HE-pro-infu-SalI-R1 | gcccttgctcaccatgtcgacCGCTTATTTTTCGGAACTGCG | |
| 1300GFP-SaMTP8.1-NHE-pro-infu-EcoRI-F1 | tatgaccatgattacgaattcCAAGGTGATGAGCGTTCA | |
| 1300GFP-SaMTP8.1-NHE-pro-infu-SalI-R1 | gcccttgctcaccatgtcgacCGCTTATTTTTTGGTACTGCG | |
| 1300GFP-SaMTP8.1-HE/NHE-GFP-infu-KpnI-F1 | gacgagctcggtaccATGGGGTTGAGAAACGGAG | Subcellular localization |
| 1300GFP-SaMTP8.1-HE/NHE-GFP-infu-SalI-R1 | gctcaccatgtcgacCTGACTGTTGGGAAGTCTGCT | |
| pYES2-SaMTP8.1-HE/NHE-infu-EcoRI-F1 | ccgccagtgtgctggATGGGGTTGAGAAACGGAG | Yeast expression |
| pYES2-SaMTP8.1-HE/NHE-infu-XbaI-R1 | gatgcggccctctagCTACTGACTGTTGGGAAGTCTG | |
| pPZP-OsMTP8.1-pro-infu-KpnI-F1 | gggcgaattgggtacCTGTGCATGGAGTGTGCAAG | Construction of complementation lines in rice |
| pPZP-OsMTP8.1-pro-R1 | CTCCGTTTCTCAACCCCATcgaatcacctctctcctttct | |
| pPZP-SaMTP8.1-HE-cds-F1 | agaaaggagagaggtgattcgATGGGGTTGAGAAACGGAG | |
| pPZP-SaMTP8.1-HE-cds-infu-HindIII-R1 | attcgatatcaagctCTActtgtcatcgtcgtccttgtaatcCTGAGTGTTGGGAAGTCTG | |
| pPZP-SaMTP8.1-HE-pro-infu-KpnI-F1 | gggcgaattgggtacCAAGGTGGCGAGTGTTCA | Construction of over-expression lines in Arabidopsis |
| pPZP-SaMTP8.1-HE-cds-infu-HindIII-R1 | attcgatatcaagctCTActtgtcatcgtcgtccttgtaatcCTGAGTGTTGGGAAGTCTG | |

## Slide 2
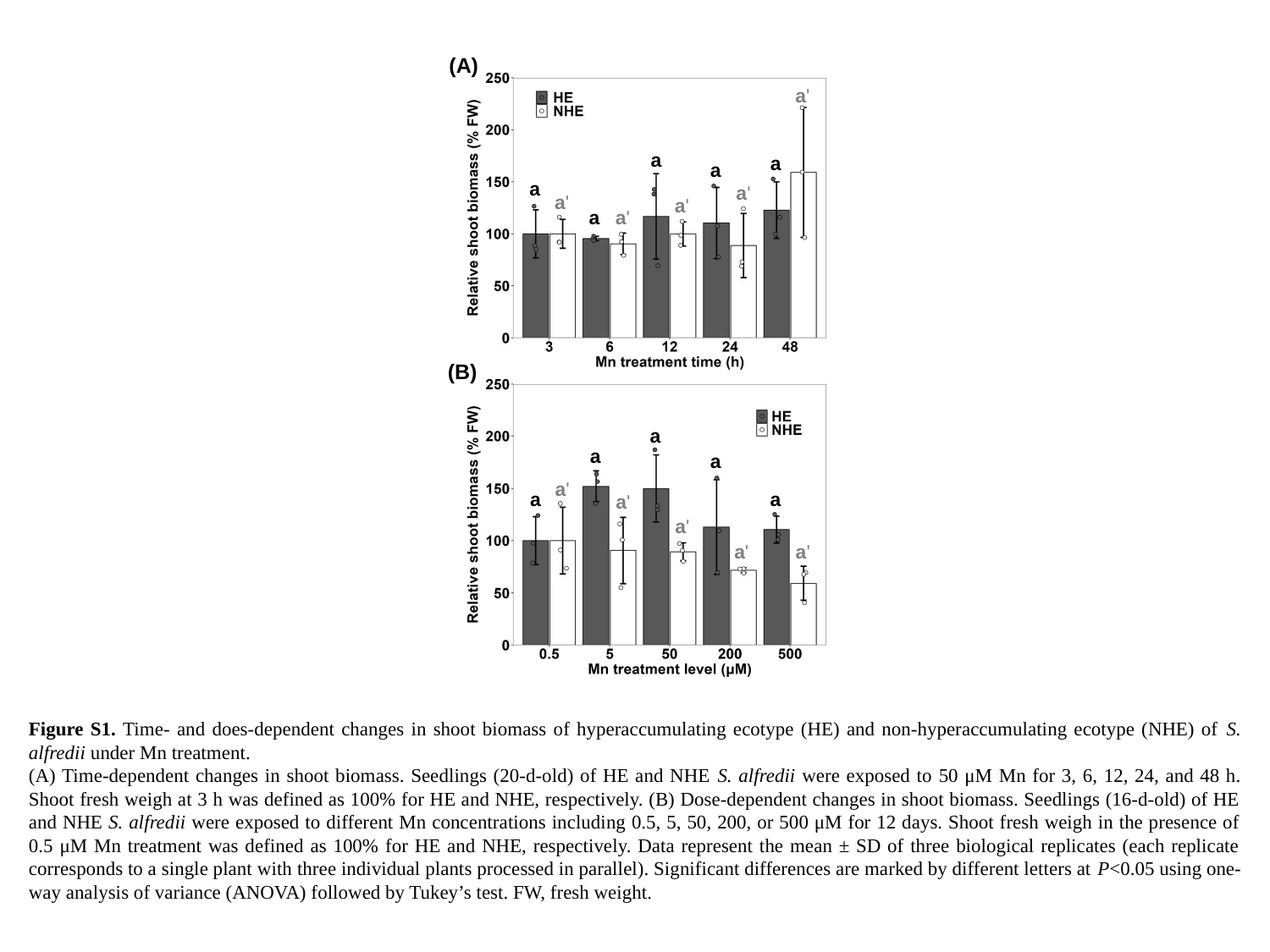

(A)
a'
a
a
a
a
a'
a'
a'
a'
a
(B)
a
a
a
a'
a
a
a'
a'
a'
a'
Figure S1. Time- and does-dependent changes in shoot biomass of hyperaccumulating ecotype (HE) and non-hyperaccumulating ecotype (NHE) of S. alfredii under Mn treatment.
(A) Time-dependent changes in shoot biomass. Seedlings (20-d-old) of HE and NHE S. alfredii were exposed to 50 μM Mn for 3, 6, 12, 24, and 48 h. Shoot fresh weigh at 3 h was defined as 100% for HE and NHE, respectively. (B) Dose-dependent changes in shoot biomass. Seedlings (16-d-old) of HE and NHE S. alfredii were exposed to different Mn concentrations including 0.5, 5, 50, 200, or 500 μM for 12 days. Shoot fresh weigh in the presence of 0.5 μM Mn treatment was defined as 100% for HE and NHE, respectively. Data represent the mean ± SD of three biological replicates (each replicate corresponds to a single plant with three individual plants processed in parallel). Significant differences are marked by different letters at P<0.05 using one-way analysis of variance (ANOVA) followed by Tukey’s test. FW, fresh weight.

## Slide 3
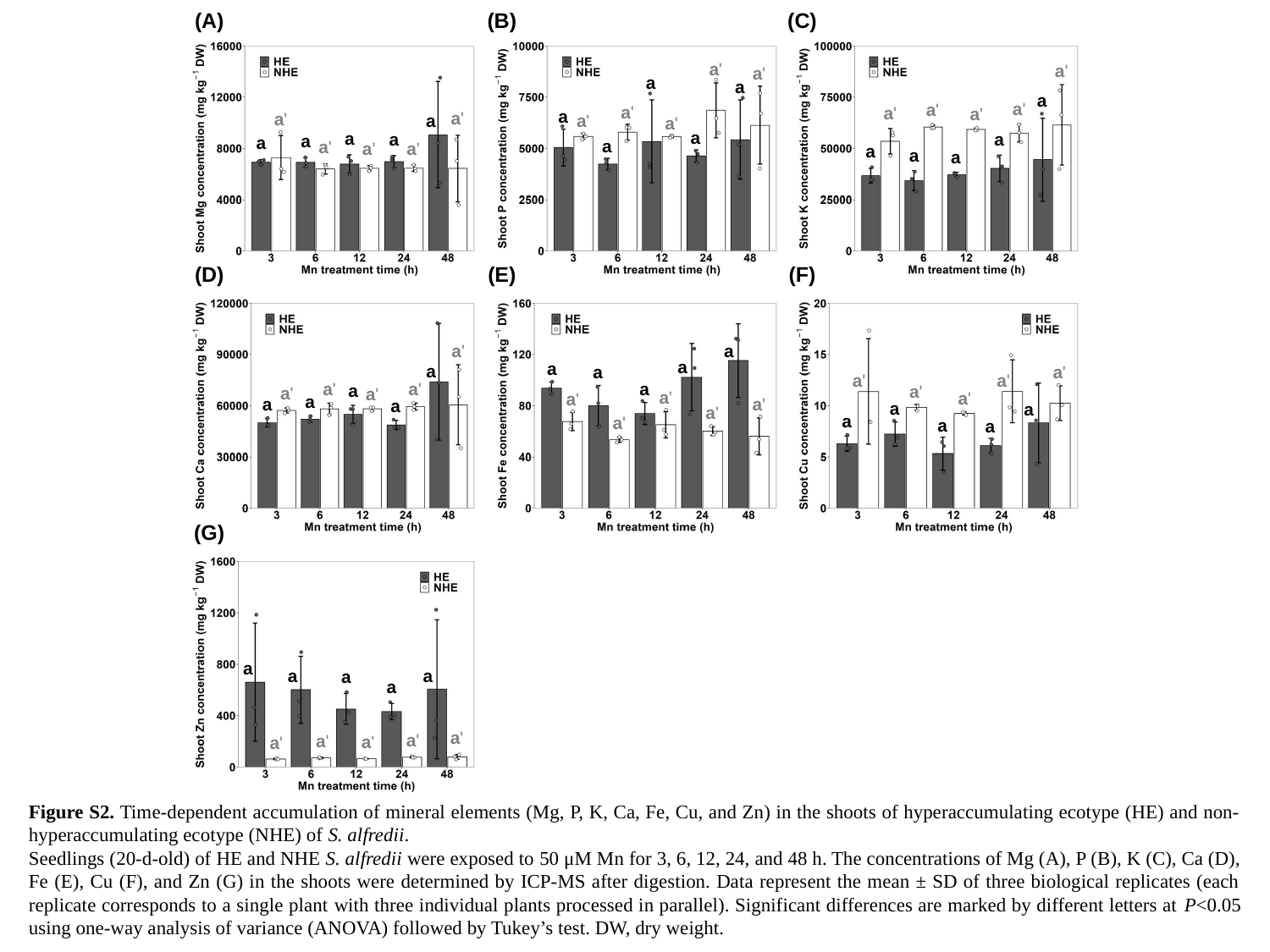

(A)
(B)
(C)
a'
a'
a
a
a'
a
a'
a'
a
a
a'
a
a'
a'
a'
a'
a
a
a
a
a'
a'
a
a
a
a
a
a'
a'
a'
(D)
(E)
(F)
a'
a
a'
a'
a
a'
a'
a
a
a
a
a
a
a
a
a'
a'
a'
a'
a'
a'
a'
a'
a'
a'
a
a
a
a
a
(G)
a
a
a
a
a
a'
a'
a'
a'
a'
Figure S2. Time-dependent accumulation of mineral elements (Mg, P, K, Ca, Fe, Cu, and Zn) in the shoots of hyperaccumulating ecotype (HE) and non-hyperaccumulating ecotype (NHE) of S. alfredii.
Seedlings (20-d-old) of HE and NHE S. alfredii were exposed to 50 μM Mn for 3, 6, 12, 24, and 48 h. The concentrations of Mg (A), P (B), K (C), Ca (D), Fe (E), Cu (F), and Zn (G) in the shoots were determined by ICP-MS after digestion. Data represent the mean ± SD of three biological replicates (each replicate corresponds to a single plant with three individual plants processed in parallel). Significant differences are marked by different letters at P<0.05 using one-way analysis of variance (ANOVA) followed by Tukey’s test. DW, dry weight.

## Slide 4
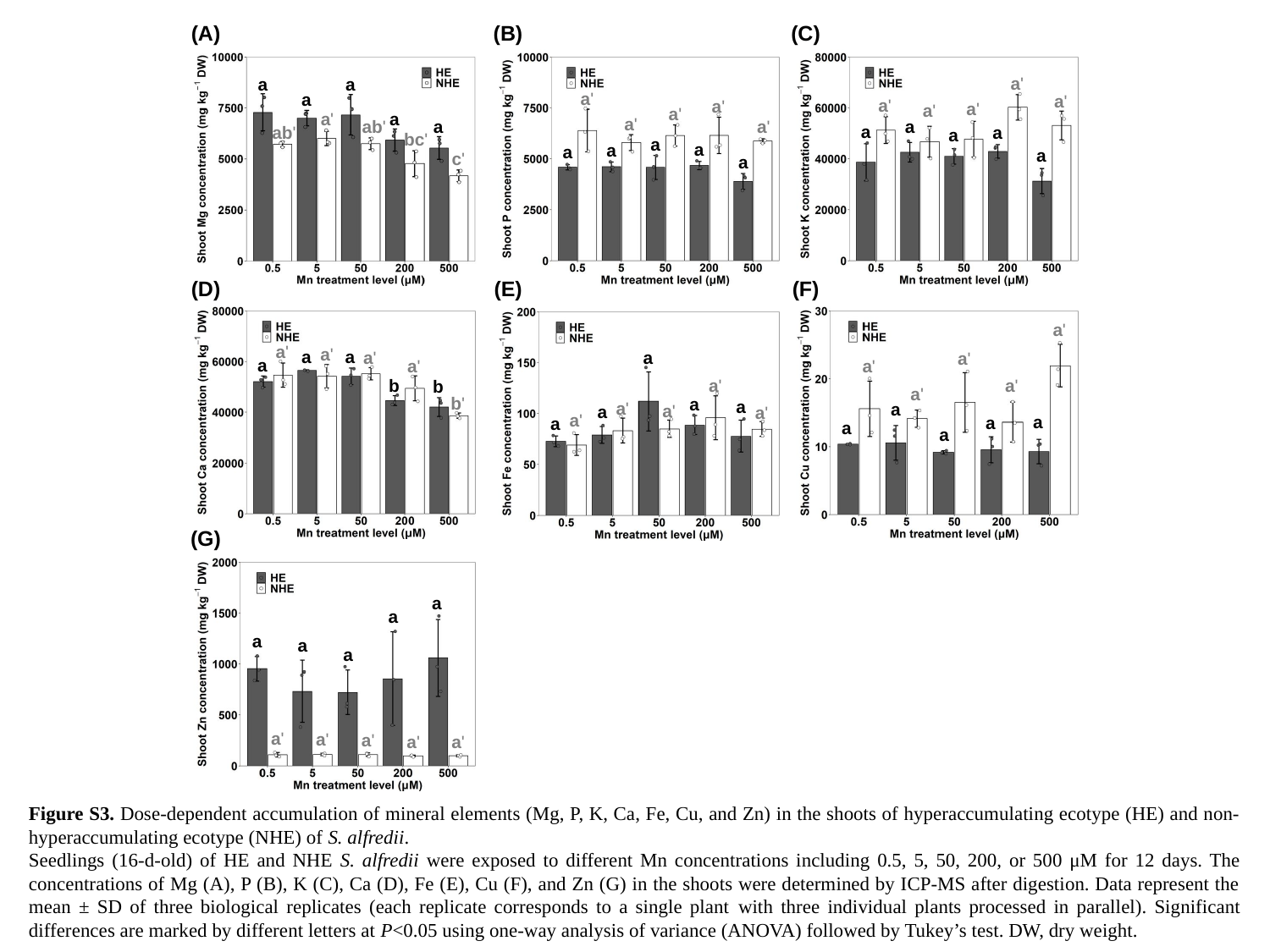

(A)
(B)
(C)
a'
a
a
a'
a
a'
a'
a'
a'
a'
a'
a
a'
a'
ab'
a
a
a'
a
a
ab'
a
bc'
a
a
a
a
a
c'
a
(D)
(E)
(F)
a'
a'
a'
a
a
a
a'
a'
a
a'
a'
b
a'
a'
b
a'
b'
a
a
a'
a
a'
a
a'
a'
a
a
a
a
a
(G)
a
a
a
a
a
a'
a'
a'
a'
a'
Figure S3. Dose-dependent accumulation of mineral elements (Mg, P, K, Ca, Fe, Cu, and Zn) in the shoots of hyperaccumulating ecotype (HE) and non-hyperaccumulating ecotype (NHE) of S. alfredii.
Seedlings (16-d-old) of HE and NHE S. alfredii were exposed to different Mn concentrations including 0.5, 5, 50, 200, or 500 μM for 12 days. The concentrations of Mg (A), P (B), K (C), Ca (D), Fe (E), Cu (F), and Zn (G) in the shoots were determined by ICP-MS after digestion. Data represent the mean ± SD of three biological replicates (each replicate corresponds to a single plant with three individual plants processed in parallel). Significant differences are marked by different letters at P<0.05 using one-way analysis of variance (ANOVA) followed by Tukey’s test. DW, dry weight.

## Slide 5
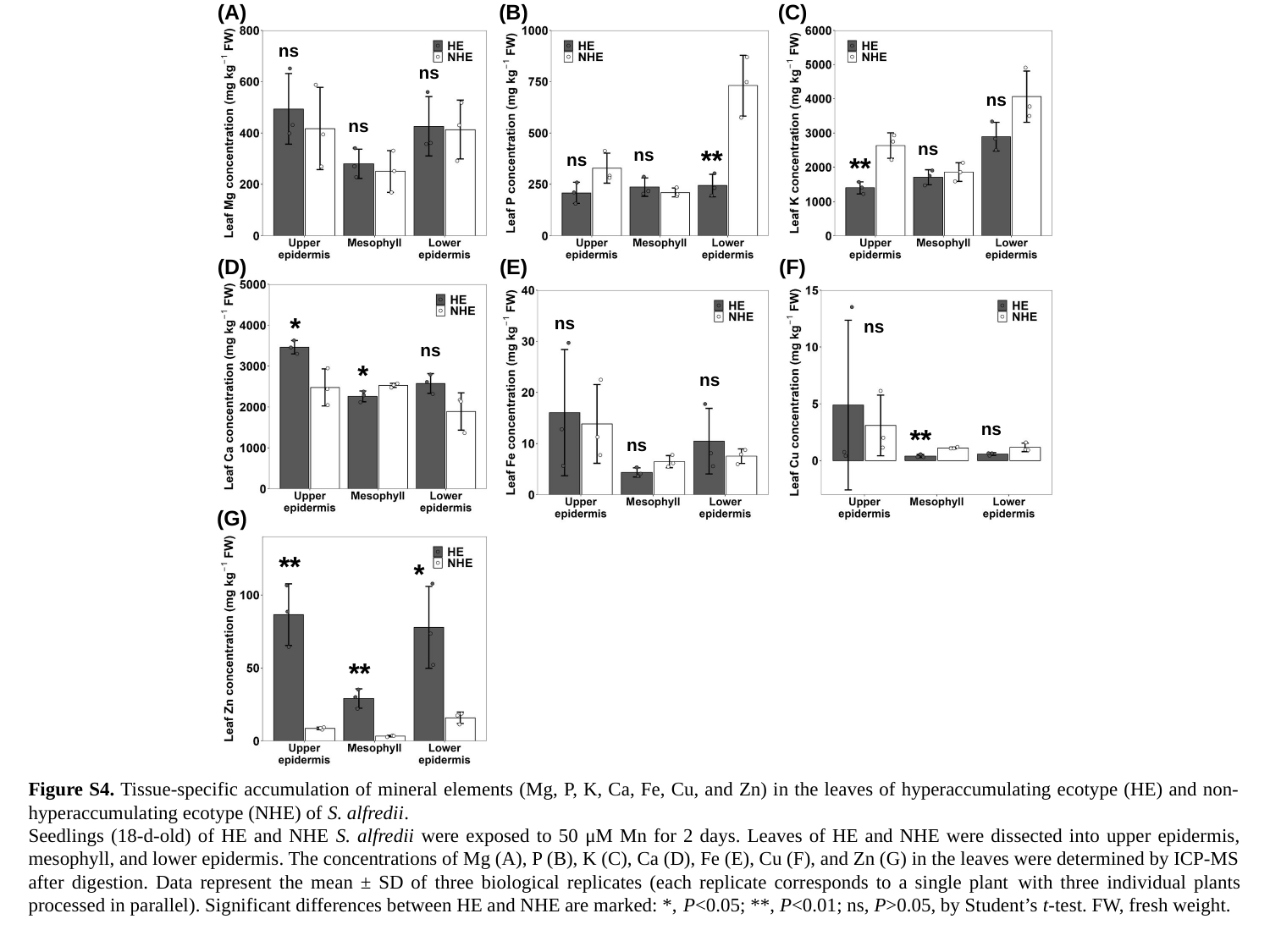

(A)
(B)
(C)
ns
ns
ns
**
ns
ns
ns
ns
**
(D)
(E)
(F)
*
ns
*
ns
ns
ns
ns
ns
**
(G)
**
*
**
Figure S4. Tissue-specific accumulation of mineral elements (Mg, P, K, Ca, Fe, Cu, and Zn) in the leaves of hyperaccumulating ecotype (HE) and non-hyperaccumulating ecotype (NHE) of S. alfredii.
Seedlings (18-d-old) of HE and NHE S. alfredii were exposed to 50 μM Mn for 2 days. Leaves of HE and NHE were dissected into upper epidermis, mesophyll, and lower epidermis. The concentrations of Mg (A), P (B), K (C), Ca (D), Fe (E), Cu (F), and Zn (G) in the leaves were determined by ICP-MS after digestion. Data represent the mean ± SD of three biological replicates (each replicate corresponds to a single plant with three individual plants processed in parallel). Significant differences between HE and NHE are marked: *, P<0.05; **, P<0.01; ns, P>0.05, by Student’s t-test. FW, fresh weight.

## Slide 6
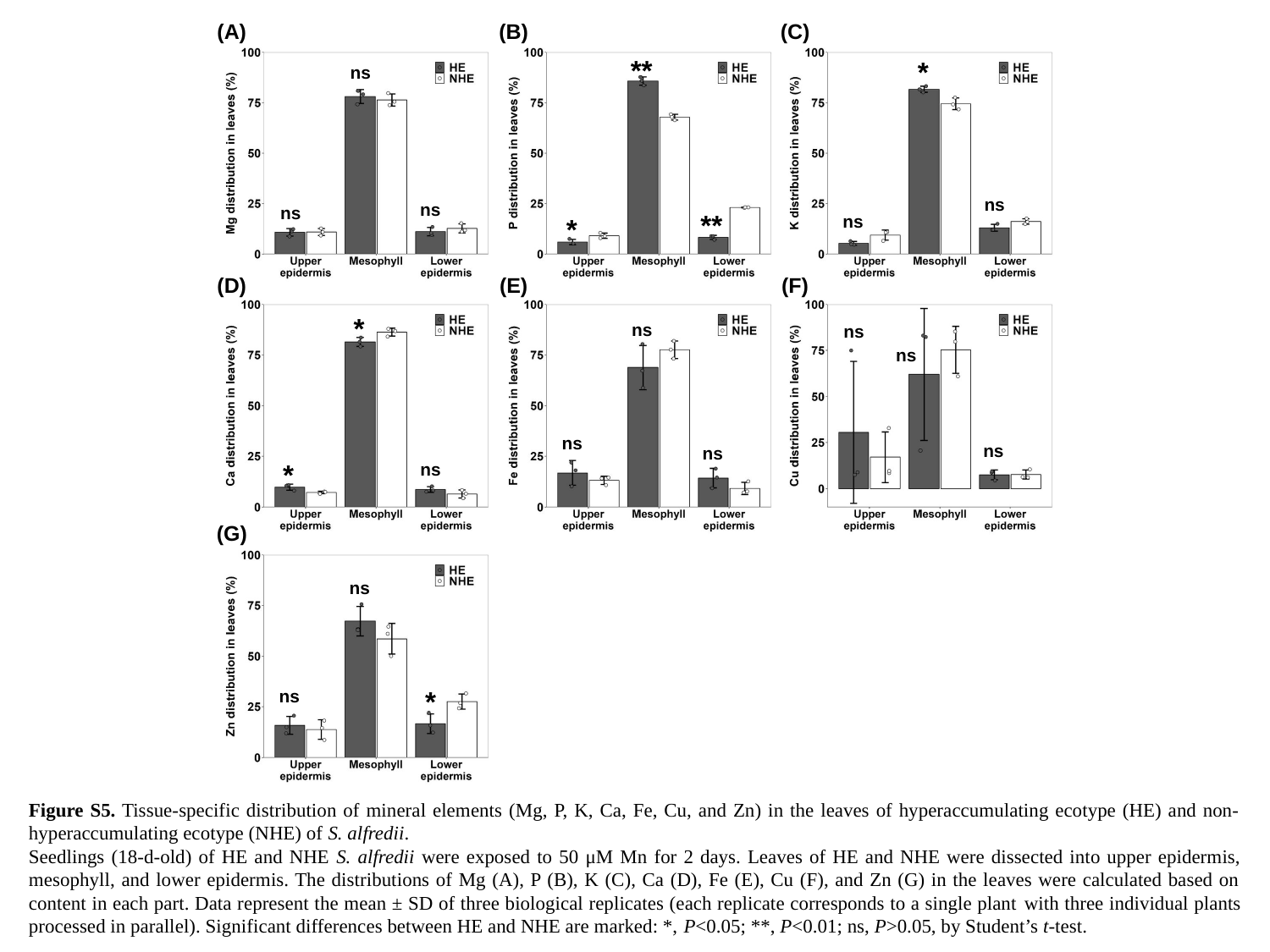

(A)
(B)
(C)
ns
ns
ns
*
ns
ns
**
**
*
(D)
(E)
(F)
*
*
ns
ns
ns
ns
ns
ns
ns
(G)
ns
*
ns
Figure S5. Tissue-specific distribution of mineral elements (Mg, P, K, Ca, Fe, Cu, and Zn) in the leaves of hyperaccumulating ecotype (HE) and non-hyperaccumulating ecotype (NHE) of S. alfredii.
Seedlings (18-d-old) of HE and NHE S. alfredii were exposed to 50 μM Mn for 2 days. Leaves of HE and NHE were dissected into upper epidermis, mesophyll, and lower epidermis. The distributions of Mg (A), P (B), K (C), Ca (D), Fe (E), Cu (F), and Zn (G) in the leaves were calculated based on content in each part. Data represent the mean ± SD of three biological replicates (each replicate corresponds to a single plant with three individual plants processed in parallel). Significant differences between HE and NHE are marked: *, P<0.05; **, P<0.01; ns, P>0.05, by Student’s t-test.

## Slide 7
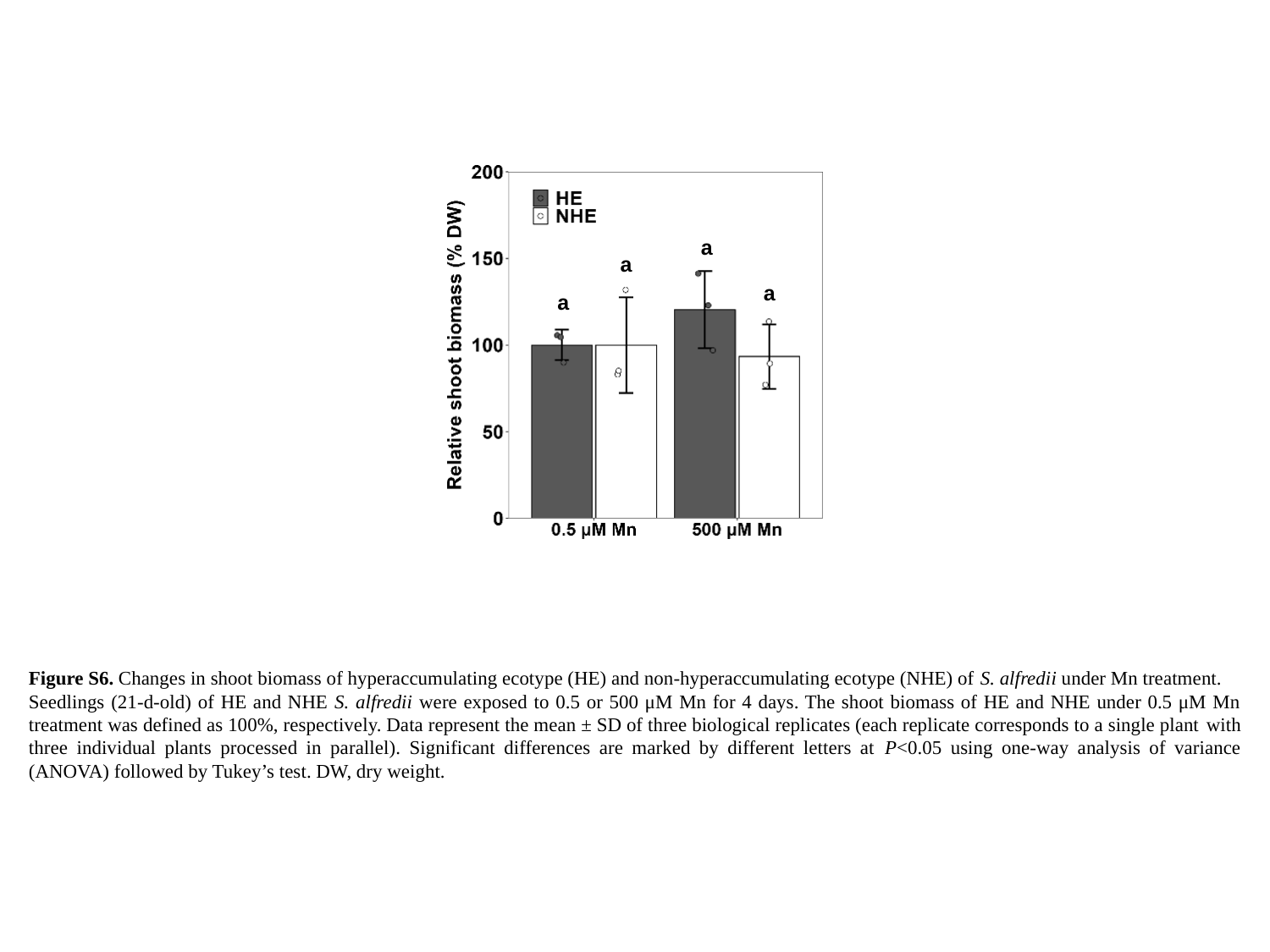

a
a
a
a
Figure S6. Changes in shoot biomass of hyperaccumulating ecotype (HE) and non-hyperaccumulating ecotype (NHE) of S. alfredii under Mn treatment.
Seedlings (21-d-old) of HE and NHE S. alfredii were exposed to 0.5 or 500 μM Mn for 4 days. The shoot biomass of HE and NHE under 0.5 μM Mn treatment was defined as 100%, respectively. Data represent the mean ± SD of three biological replicates (each replicate corresponds to a single plant with three individual plants processed in parallel). Significant differences are marked by different letters at P<0.05 using one-way analysis of variance (ANOVA) followed by Tukey’s test. DW, dry weight.

## Slide 8
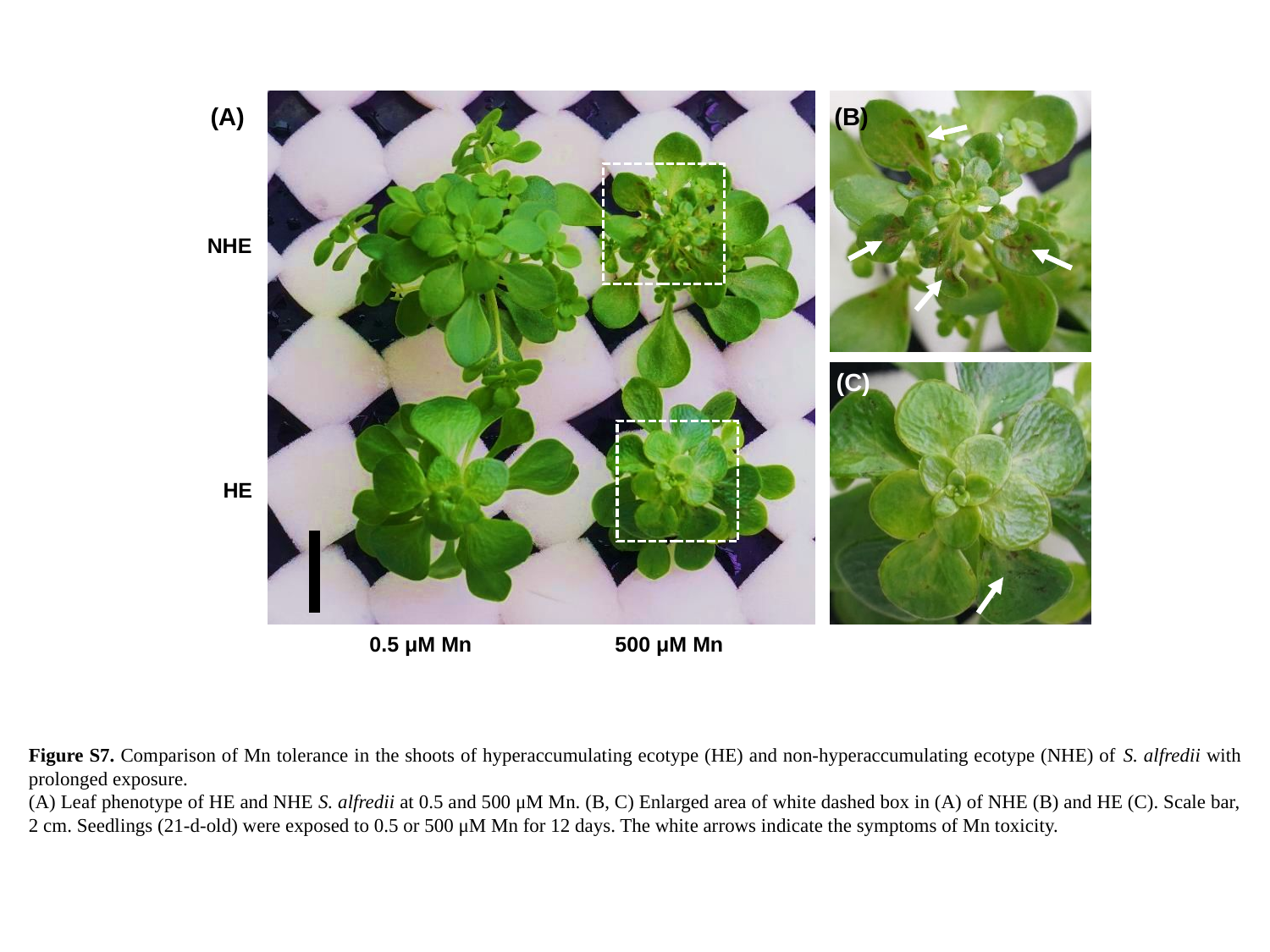

(A)
(B)
NHE
(C)
HE
0.5 μM Mn
500 μM Mn
Figure S7. Comparison of Mn tolerance in the shoots of hyperaccumulating ecotype (HE) and non-hyperaccumulating ecotype (NHE) of S. alfredii with prolonged exposure.
(A) Leaf phenotype of HE and NHE S. alfredii at 0.5 and 500 μM Mn. (B, C) Enlarged area of white dashed box in (A) of NHE (B) and HE (C). Scale bar, 2 cm. Seedlings (21-d-old) were exposed to 0.5 or 500 μM Mn for 12 days. The white arrows indicate the symptoms of Mn toxicity.

## Slide 9
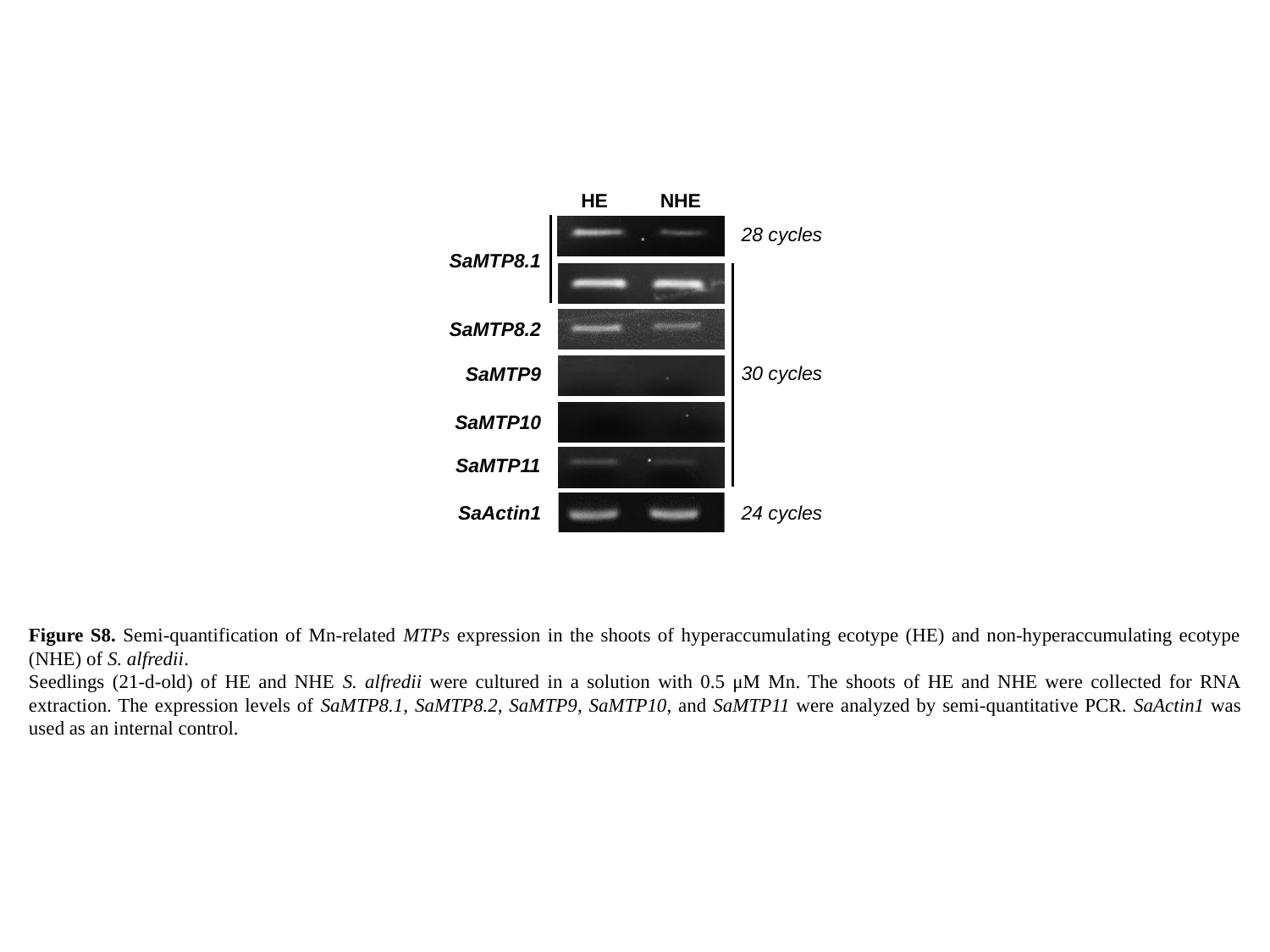

HE
NHE
28 cycles
SaMTP8.1
SaMTP8.2
30 cycles
SaMTP9
SaMTP10
SaMTP11
SaActin1
24 cycles
Figure S8. Semi-quantification of Mn-related MTPs expression in the shoots of hyperaccumulating ecotype (HE) and non-hyperaccumulating ecotype (NHE) of S. alfredii.
Seedlings (21-d-old) of HE and NHE S. alfredii were cultured in a solution with 0.5 μM Mn. The shoots of HE and NHE were collected for RNA extraction. The expression levels of SaMTP8.1, SaMTP8.2, SaMTP9, SaMTP10, and SaMTP11 were analyzed by semi-quantitative PCR. SaActin1 was used as an internal control.

## Slide 10
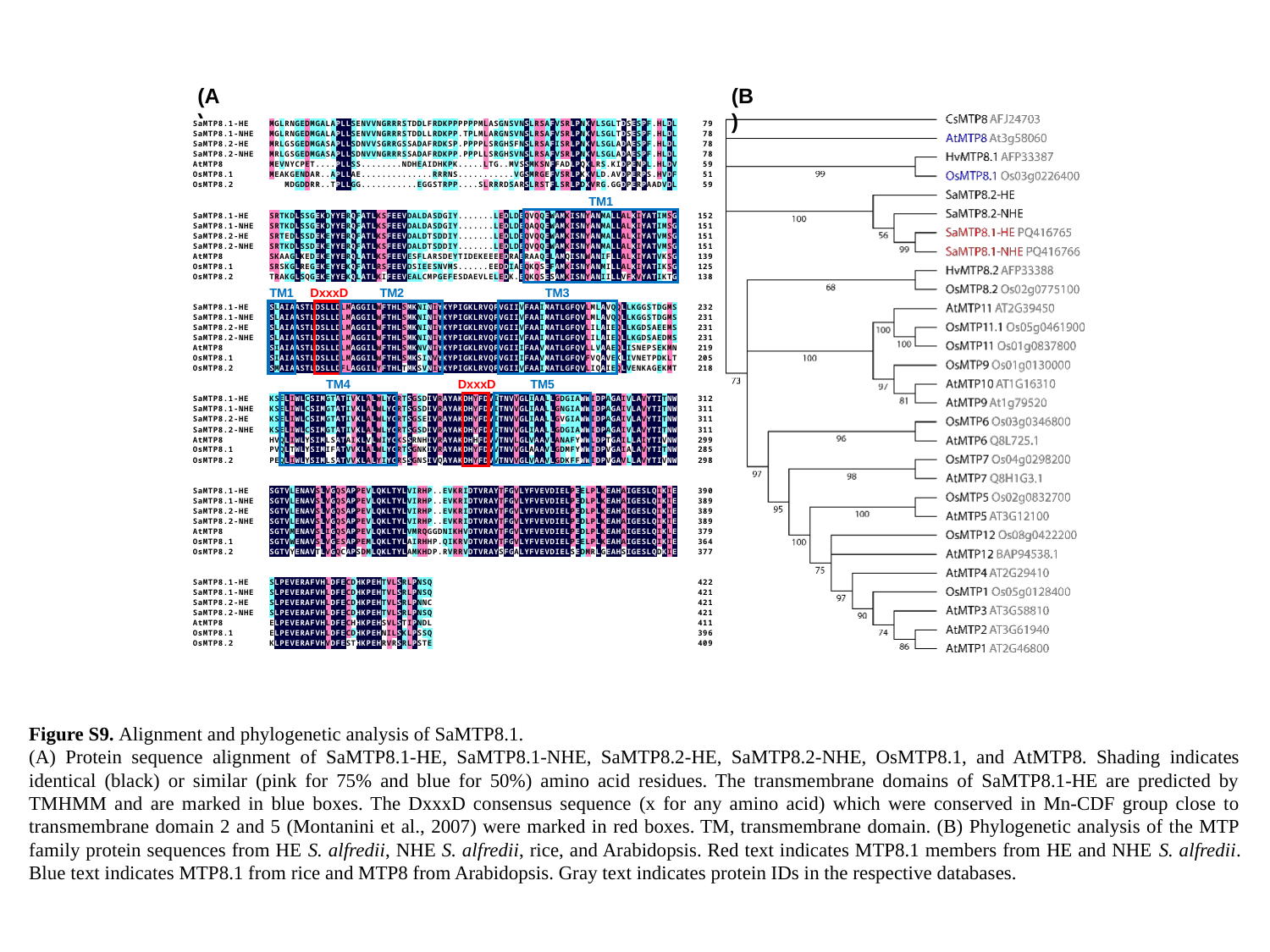

(A)
(B)
TM1
TM1
DxxxD
TM2
TM3
TM4
DxxxD
TM5
Figure S9. Alignment and phylogenetic analysis of SaMTP8.1.
(A) Protein sequence alignment of SaMTP8.1-HE, SaMTP8.1-NHE, SaMTP8.2-HE, SaMTP8.2-NHE, OsMTP8.1, and AtMTP8. Shading indicates identical (black) or similar (pink for 75% and blue for 50%) amino acid residues. The transmembrane domains of SaMTP8.1-HE are predicted by TMHMM and are marked in blue boxes. The DxxxD consensus sequence (x for any amino acid) which were conserved in Mn-CDF group close to transmembrane domain 2 and 5 (Montanini et al., 2007) were marked in red boxes. TM, transmembrane domain. (B) Phylogenetic analysis of the MTP family protein sequences from HE S. alfredii, NHE S. alfredii, rice, and Arabidopsis. Red text indicates MTP8.1 members from HE and NHE S. alfredii. Blue text indicates MTP8.1 from rice and MTP8 from Arabidopsis. Gray text indicates protein IDs in the respective databases.

## Slide 11
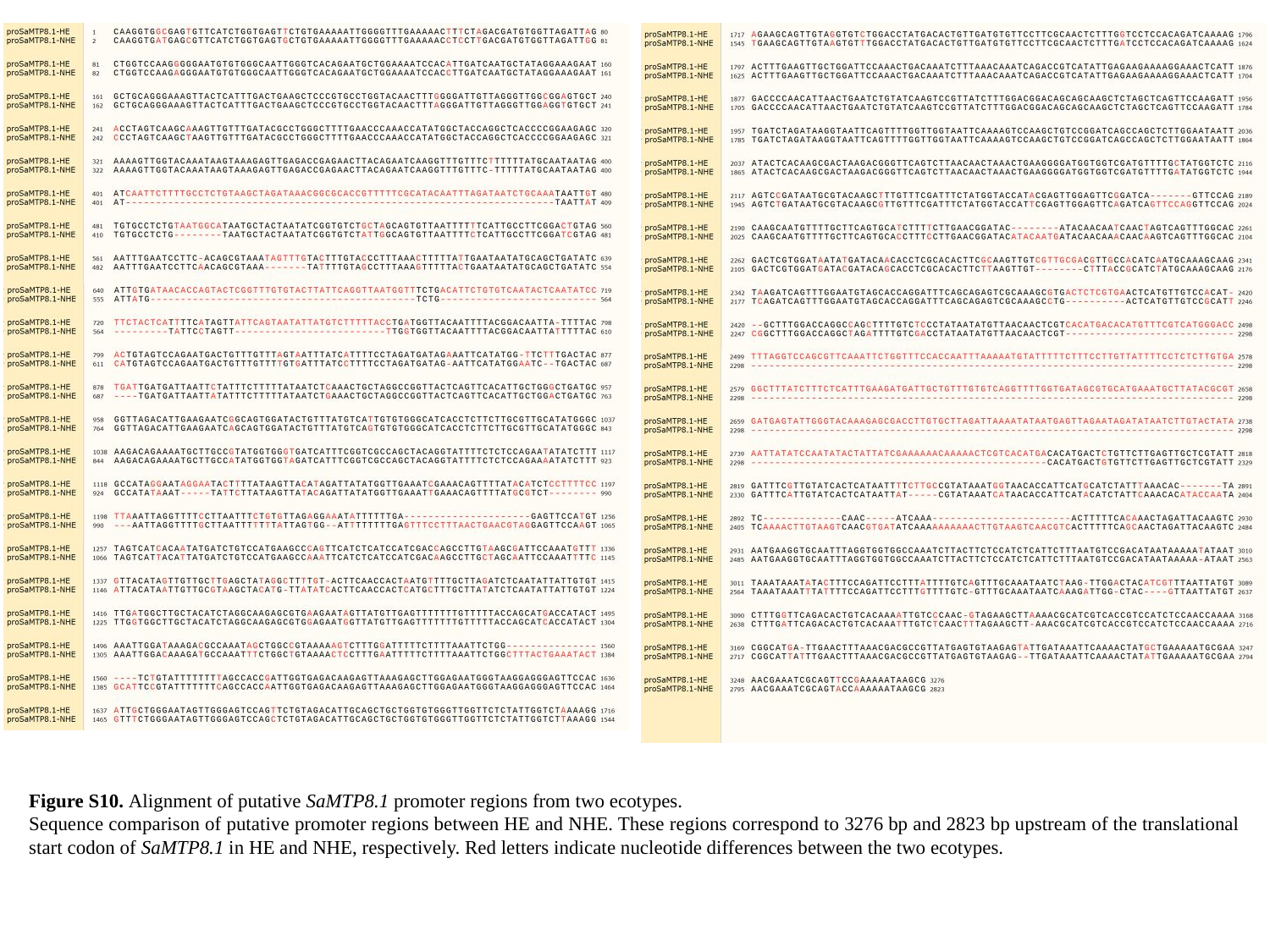

Figure S10. Alignment of putative SaMTP8.1 promoter regions from two ecotypes.
Sequence comparison of putative promoter regions between HE and NHE. These regions correspond to 3276 bp and 2823 bp upstream of the translational start codon of SaMTP8.1 in HE and NHE, respectively. Red letters indicate nucleotide differences between the two ecotypes.

## Slide 12
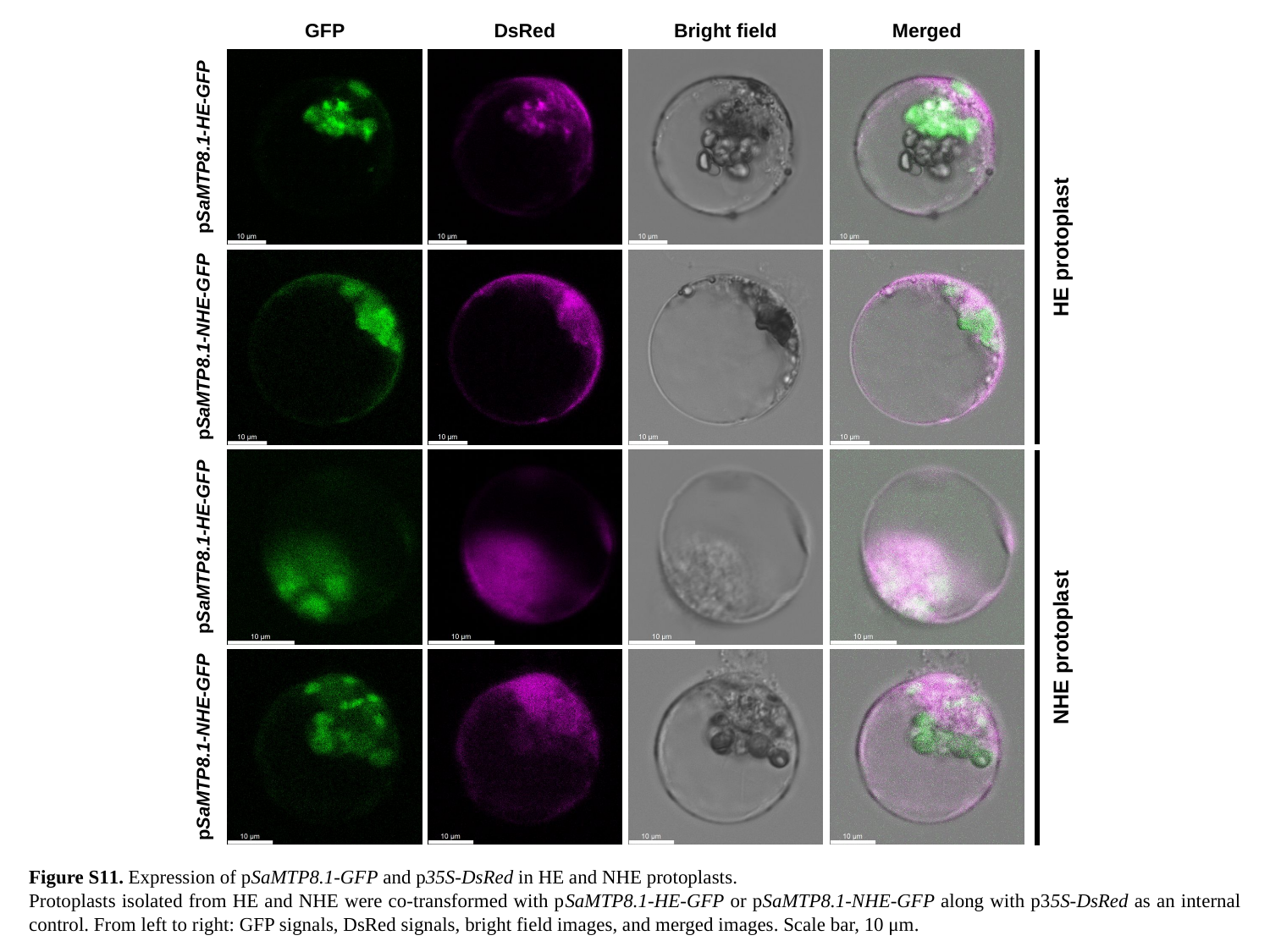

GFP
DsRed
Bright field
Merged
pSaMTP8.1-HE-GFP
HE protoplast
pSaMTP8.1-NHE-GFP
pSaMTP8.1-HE-GFP
NHE protoplast
pSaMTP8.1-NHE-GFP
Figure S11. Expression of pSaMTP8.1-GFP and p35S-DsRed in HE and NHE protoplasts.
Protoplasts isolated from HE and NHE were co-transformed with pSaMTP8.1-HE-GFP or pSaMTP8.1-NHE-GFP along with p35S-DsRed as an internal control. From left to right: GFP signals, DsRed signals, bright field images, and merged images. Scale bar, 10 μm.

## Slide 13
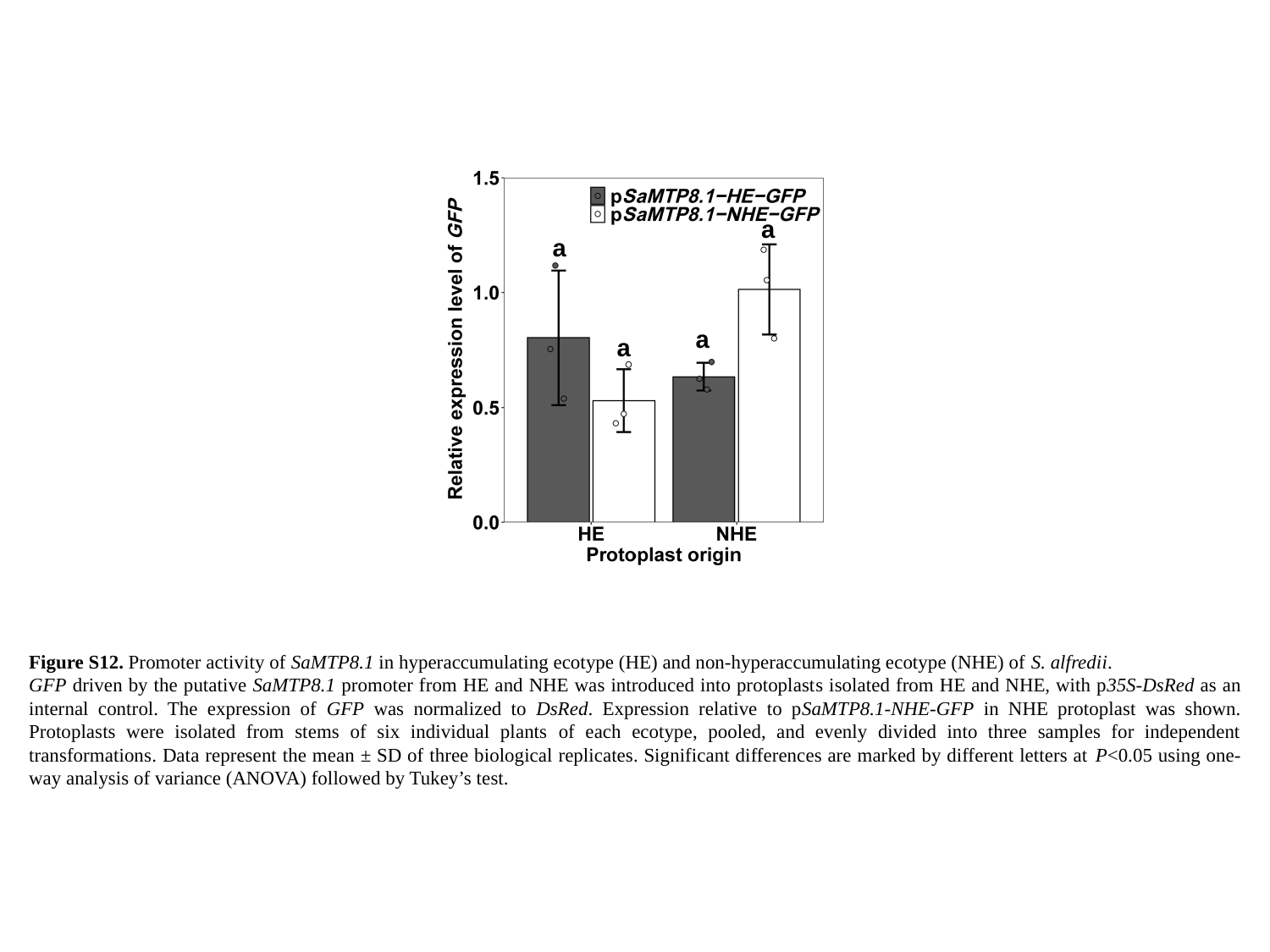

a
a
a
a
Figure S12. Promoter activity of SaMTP8.1 in hyperaccumulating ecotype (HE) and non-hyperaccumulating ecotype (NHE) of S. alfredii.
GFP driven by the putative SaMTP8.1 promoter from HE and NHE was introduced into protoplasts isolated from HE and NHE, with p35S-DsRed as an internal control. The expression of GFP was normalized to DsRed. Expression relative to pSaMTP8.1-NHE-GFP in NHE protoplast was shown. Protoplasts were isolated from stems of six individual plants of each ecotype, pooled, and evenly divided into three samples for independent transformations. Data represent the mean ± SD of three biological replicates. Significant differences are marked by different letters at P<0.05 using one-way analysis of variance (ANOVA) followed by Tukey’s test.

## Slide 14
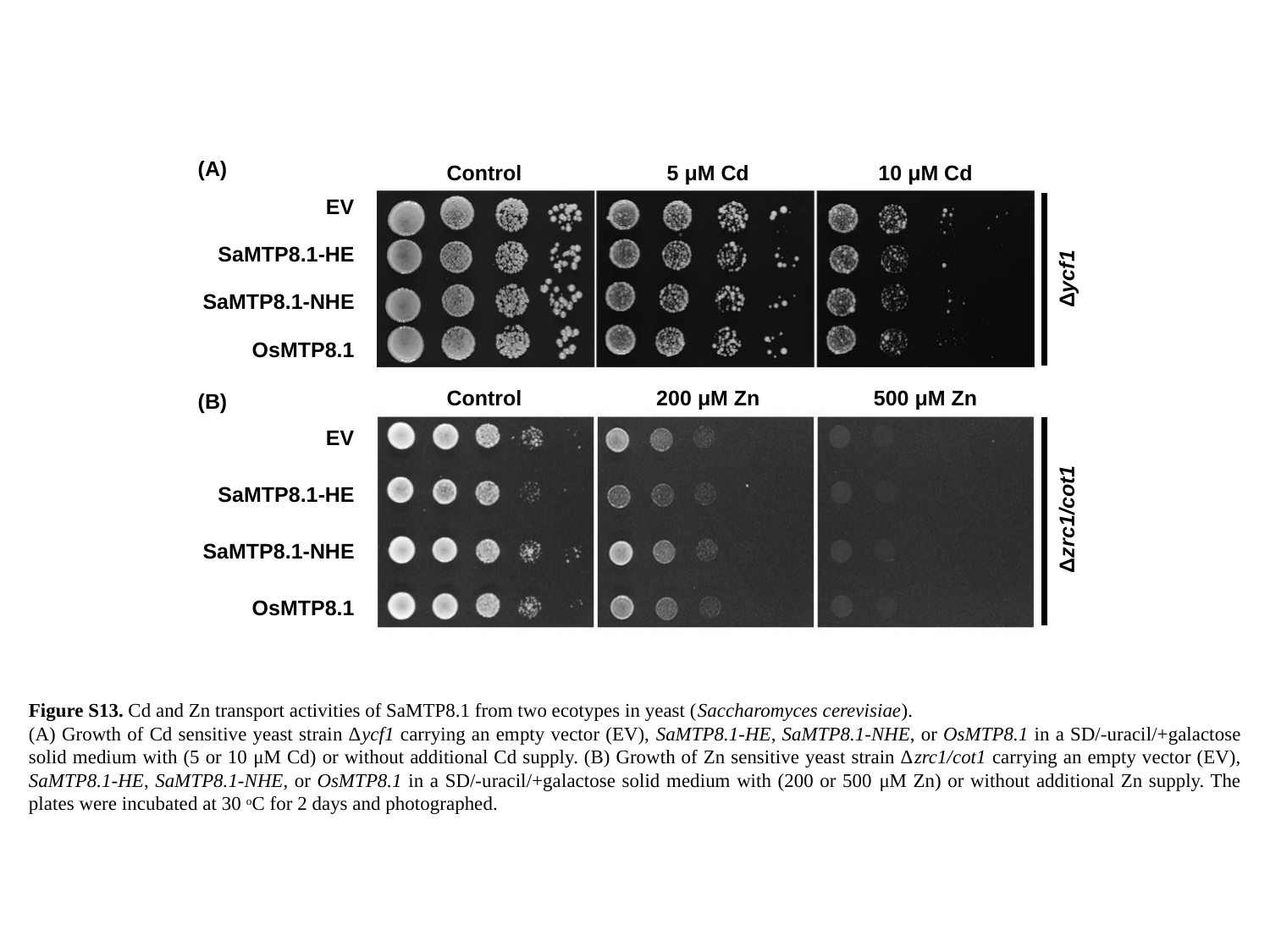

(A)
Control
5 μM Cd
10 μM Cd
EV
SaMTP8.1-HE
Δycf1
SaMTP8.1-NHE
OsMTP8.1
Control
200 μM Zn
500 μM Zn
(B)
EV
SaMTP8.1-HE
Δzrc1/cot1
SaMTP8.1-NHE
OsMTP8.1
Figure S13. Cd and Zn transport activities of SaMTP8.1 from two ecotypes in yeast (Saccharomyces cerevisiae).
(A) Growth of Cd sensitive yeast strain Δycf1 carrying an empty vector (EV), SaMTP8.1-HE, SaMTP8.1-NHE, or OsMTP8.1 in a SD/-uracil/+galactose solid medium with (5 or 10 μM Cd) or without additional Cd supply. (B) Growth of Zn sensitive yeast strain Δzrc1/cot1 carrying an empty vector (EV), SaMTP8.1-HE, SaMTP8.1-NHE, or OsMTP8.1 in a SD/-uracil/+galactose solid medium with (200 or 500 μM Zn) or without additional Zn supply. The plates were incubated at 30 oC for 2 days and photographed.

## Slide 15
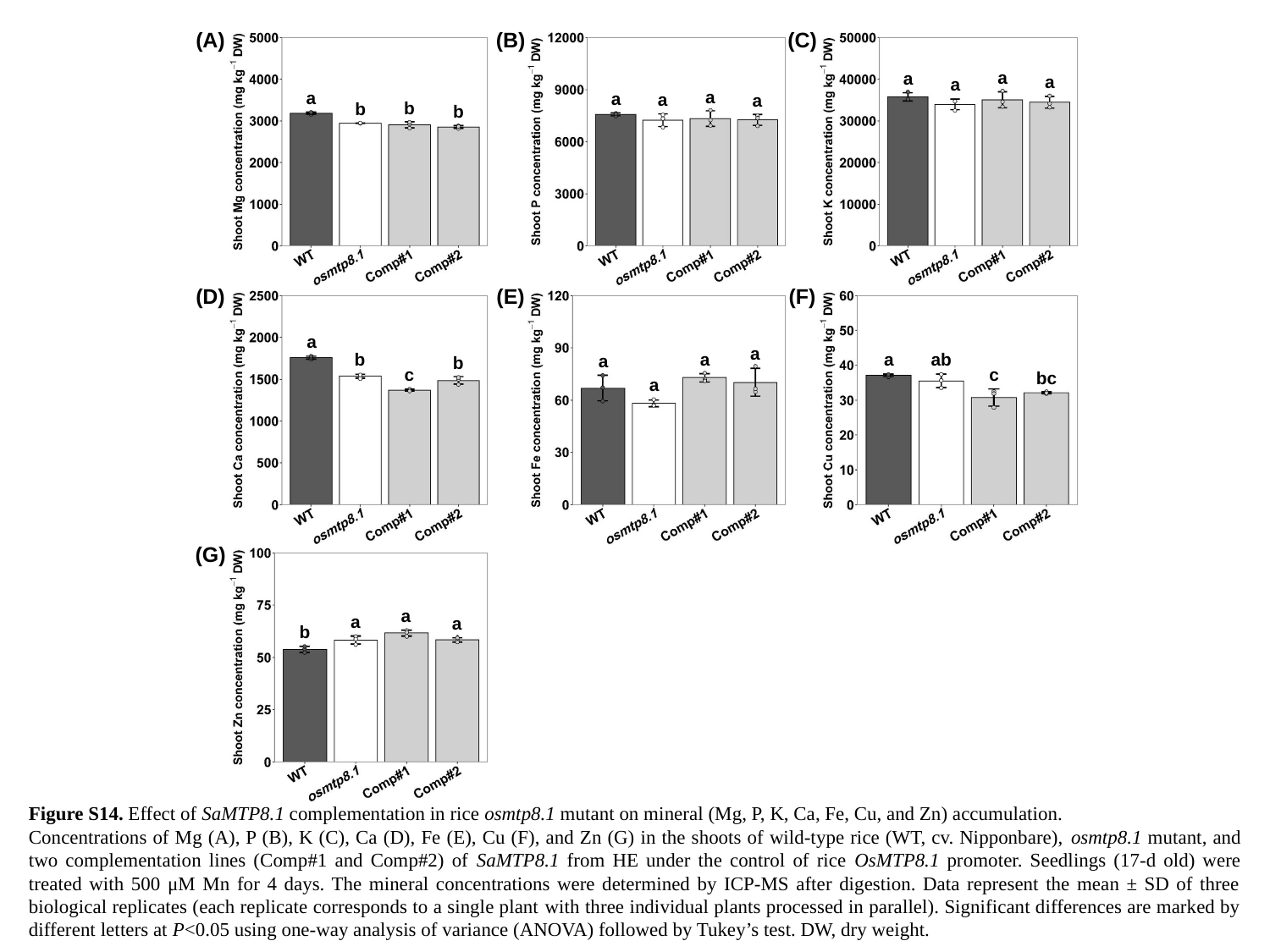

(A)
(B)
(C)
a
b
b
b
a
a
a
a
a
a
a
a
(D)
(E)
(F)
a
b
b
c
a
a
a
a
a
ab
c
bc
(G)
a
a
a
b
Figure S14. Effect of SaMTP8.1 complementation in rice osmtp8.1 mutant on mineral (Mg, P, K, Ca, Fe, Cu, and Zn) accumulation.
Concentrations of Mg (A), P (B), K (C), Ca (D), Fe (E), Cu (F), and Zn (G) in the shoots of wild-type rice (WT, cv. Nipponbare), osmtp8.1 mutant, and two complementation lines (Comp#1 and Comp#2) of SaMTP8.1 from HE under the control of rice OsMTP8.1 promoter. Seedlings (17-d old) were treated with 500 μM Mn for 4 days. The mineral concentrations were determined by ICP-MS after digestion. Data represent the mean ± SD of three biological replicates (each replicate corresponds to a single plant with three individual plants processed in parallel). Significant differences are marked by different letters at P<0.05 using one-way analysis of variance (ANOVA) followed by Tukey’s test. DW, dry weight.

## Slide 16
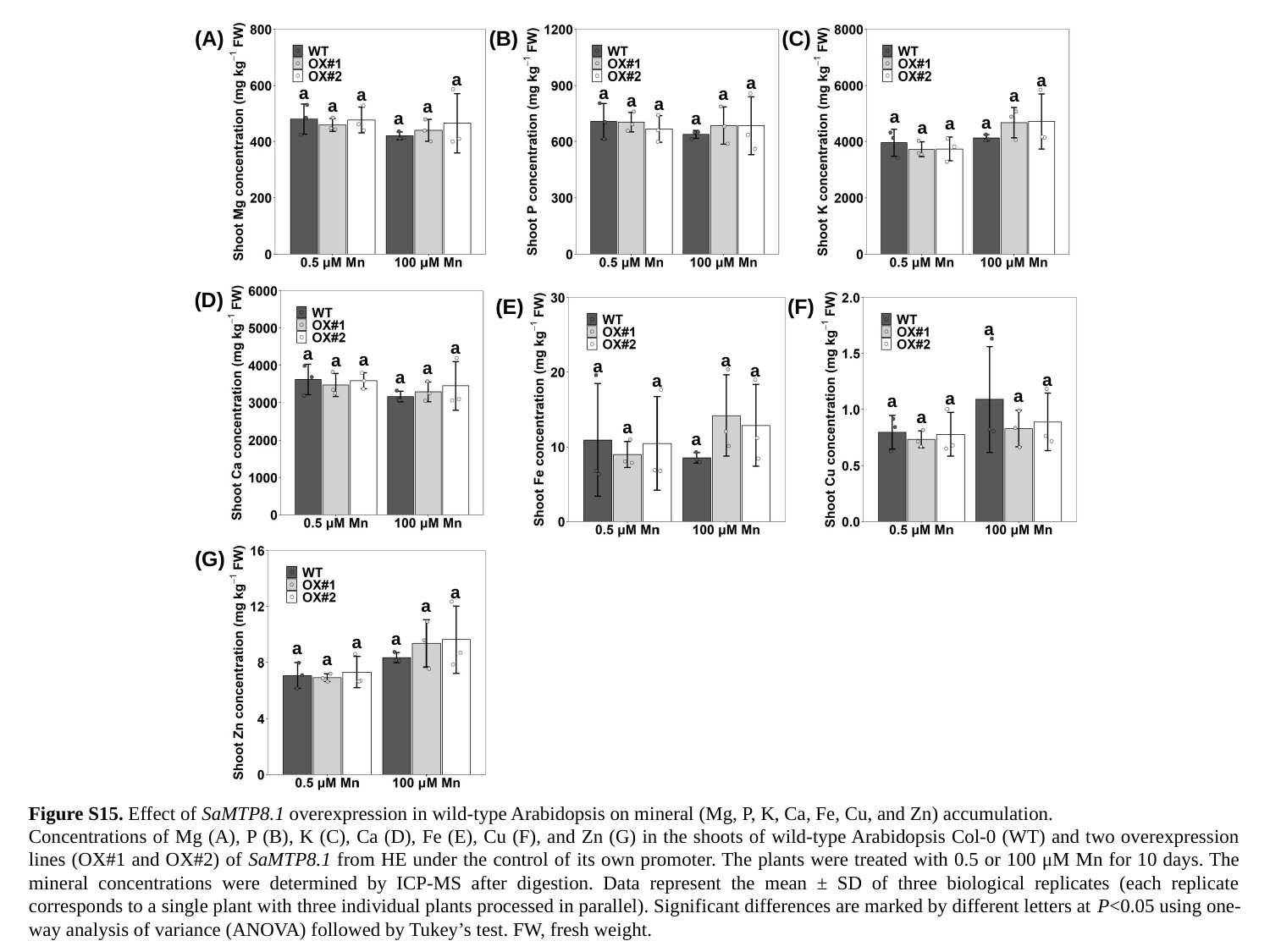

a
a
a
a
a
a
a
a
a
a
a
a
a
a
a
a
a
a
(A)
(B)
(C)
a
a
a
a
a
a
a
a
a
a
a
a
a
a
a
a
a
a
(D)
(E)
(F)
a
a
a
a
a
a
(G)
Figure S15. Effect of SaMTP8.1 overexpression in wild-type Arabidopsis on mineral (Mg, P, K, Ca, Fe, Cu, and Zn) accumulation.
Concentrations of Mg (A), P (B), K (C), Ca (D), Fe (E), Cu (F), and Zn (G) in the shoots of wild-type Arabidopsis Col-0 (WT) and two overexpression lines (OX#1 and OX#2) of SaMTP8.1 from HE under the control of its own promoter. The plants were treated with 0.5 or 100 μM Mn for 10 days. The mineral concentrations were determined by ICP-MS after digestion. Data represent the mean ± SD of three biological replicates (each replicate corresponds to a single plant with three individual plants processed in parallel). Significant differences are marked by different letters at P<0.05 using one-way analysis of variance (ANOVA) followed by Tukey’s test. FW, fresh weight.

## Slide 17
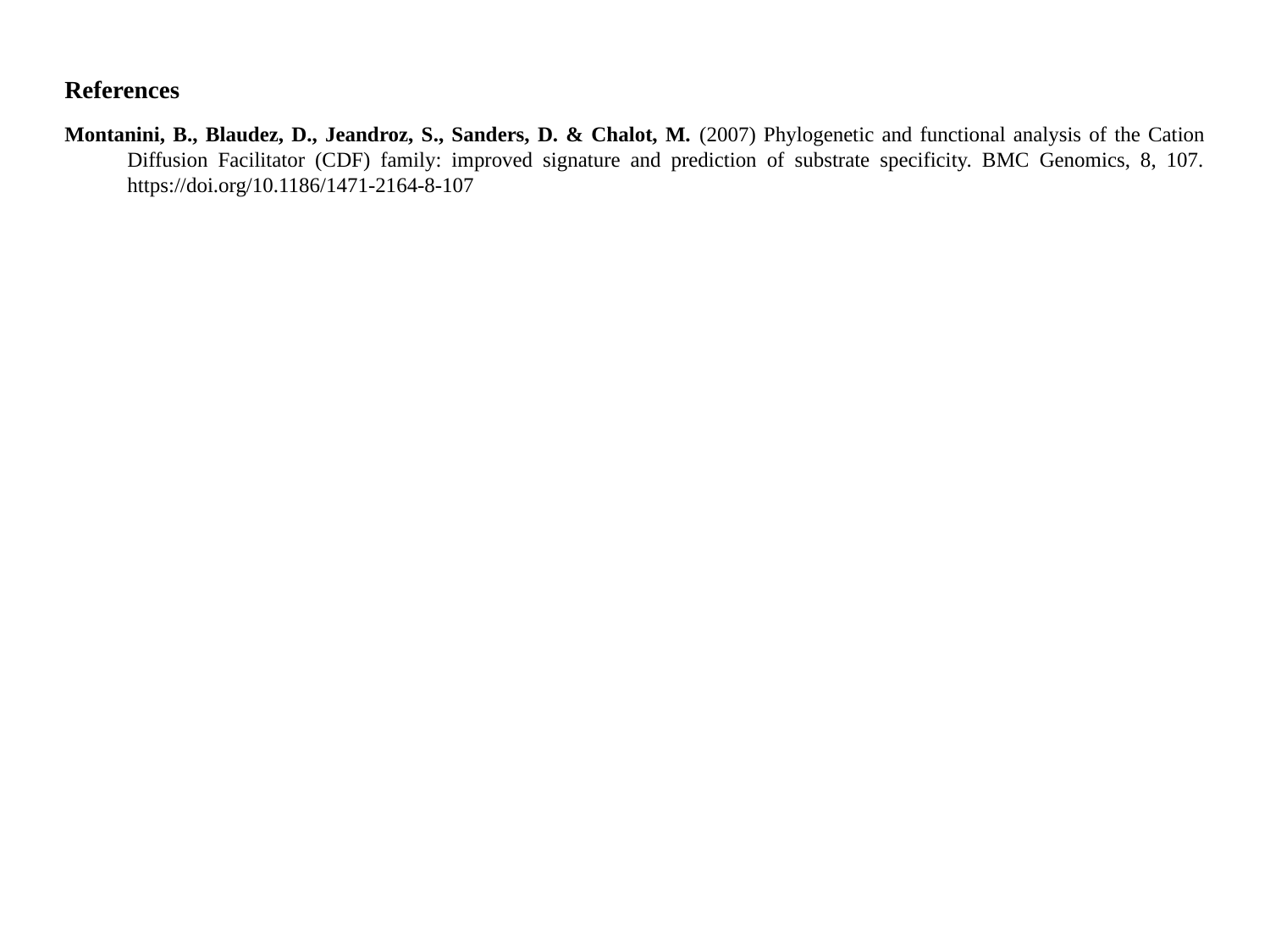

References
Montanini, B., Blaudez, D., Jeandroz, S., Sanders, D. & Chalot, M. (2007) Phylogenetic and functional analysis of the Cation Diffusion Facilitator (CDF) family: improved signature and prediction of substrate specificity. BMC Genomics, 8, 107. https://doi.org/10.1186/1471-2164-8-107
